# Clinical Application of Peripheral Blood Biomarkers for Solid Tumors

**DOI:** 10.1002/mco2.70654

**Published:** 2026-03-04

**Authors:** Xinru Tu, Mengyan Tu, Junfen Xu

**Affiliations:** ^1^ Department of Gynecologic Oncology Women's Hospital Zhejiang University School of Medicine Hangzhou Zhejiang China

**Keywords:** blood biomarkers, machine learning, noninvasive tumor detection, precision oncology

## Abstract

The growing emphasis on precision medicine in the management of solid tumors has underscored the limitations of traditional diagnostic approaches, which often lack sufficient sensitivity or rely on invasive procedures. In contrast, peripheral blood biomarkers provide a minimally invasive, dynamic, and potentially more accurate means for cancer detection and monitoring. The enhancement of detection technology has enabled the incorporation of an increasing number of biomarkers into exploratory clinical trials, which, in turn, have demonstrated immense clinical utility. However, numerous hurdles remain before these biomarkers can be applied in a real clinical setting. This review comprehensively summarizes the clinical utility of key blood‐based biomarkers, including circulating tumor cells, circulating tumor DNA, extracellular vesicles, cell‐free RNA, peripheral blood mononuclear cells, and proteins. We discuss their biological characteristics, detection methodologies, and recent advances in their clinical applications. Moreover, we highlight the emerging role of new technologies such as artificial intelligence (AI) in decoding complex data and facilitating clinical decision‐making. It is expected to establish the overarching concept of the blood biomarker panel and to understand its comparative advantages, which are essential to realize its potential in precision oncology.

## Graphical abstract

Peripheral blood biomarkers provide a minimally invasive, dynamic, and potentially more accurate means to obtain a comprehensive tumor profile. Technologies for detecting them are advancing, and with the help of artificial intelligence, they are being used more and more often for cancer diagnosis, prognostic assessment, and therapeutic monitoring.

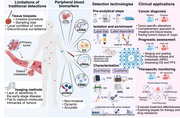

## 1 Introduction

Cancer remains a leading global health challenge with profound societal and economic implications, particularly in the context of solid tumors such as lung, colorectal, and breast cancer [1]. Characterized by the uncontrolled proliferation of abnormal cells with the potential to invade surrounding tissues and metastasize via lymphatic or hematogenous routes, solid tumors require timely diagnosis and longitudinal monitoring to achieve optimal treatment outcomes. In recent decades, the field of oncology has witnessed a transformative shift, moving from a macroscopic view centered on anatomical and histological analysis to a deeper exploration of molecular mechanisms and cellular dynamics [2]. This evolution has challenged the conventional “one‐size‐fits‐all” approaches and catalyzed the era of precision medicine, wherein treatment strategies are tailored to individual patient and tumor profiles. The advent of targeted therapies and immunotherapies has revolutionized the management of certain advanced malignancies, yielding substantial clinical benefits [3].

**FIGURE 1:**
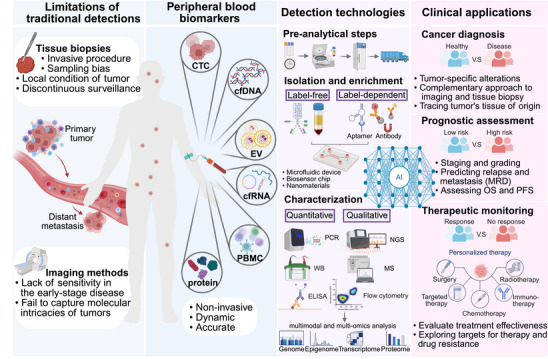
Overview of the peripheral blood biomarkers. Due to insufficient sensitivity or reliance on invasive procedures, traditional detection methods are gradually failing to meet the clinical needs of solid tumor diagnosis and treatment. In contrast, peripheral blood biomarkers provide a noninvasive, dynamic, and accurate approach to detect tumors, which are systemic and heterogeneous diseases. By isolating and characterizing these tumor‐associated substances in the circulating system, including CTCs, ctDNA, EVs, cfRNAs, PBMCs, and proteins, a comprehensive tumor profile can be obtained. This approach is poised to become a complementary or alternative tool to traditional methods in critical areas such as cancer diagnosis, prognostic assessment, and treatment monitoring. AI is also demonstrating its significance in decoding complex data and facilitating clinical decision‐making. PCR, polymerase chain reaction; ELISA, enzyme‐linked immunosorbent assay; WB, western blot; NGS, next‐generation sequencing; MS, mass spectrum. Created in https://BioRender.com.

Compared with traditional chemotherapy and radiotherapy, targeted therapies inhibit oncogenic pathways with greater specificity, minimizing collateral damage to normal tissues; and immunotherapeutic strategies—most notably immune checkpoint blockade (ICB) targeting programmed cell death protein 1 (PD‐1) or cytotoxic T lymphocyte‐associated antigen 4 (CTLA‐4)—have reactivated antitumor immunity and reshaped the treatment landscape [3]. However, variability in patient response and the emergence of drug resistance remain formidable challenges. To overcome these barriers and further personalize treatment, there is an urgent need for robust, accessible, and dynamic biomarkers to capture tumor heterogeneity, monitor disease progression, and predict therapeutic efficacy. Traditional diagnostic modalities, such as imaging and tissue biopsies, although widely adopted in clinical practice, have notable limitations (Figure 1). Imaging often lacks sensitivity in early‐stage disease and fails to capture the molecular intricacies of tumors [4]. Tissue biopsy, while providing molecular insights, is invasive and limited by sampling bias and procedural risks [5]. Moreover, it is generally impractical for continuous surveillance, especially in the absence of measurable disease after initial treatment. These limitations underscore the need for more minimally invasive, real‐time, and comprehensive diagnostic strategies.

As cancer is increasingly recognized as a systemic and heterogeneous disease, tumor‐associated components—nucleic acids, proteins, cells, and extracellular vesicles (EVs)—are shed into the bloodstream and may reflect the dynamic biological status of the tumor and its microenvironment [2]. These circulating analytes, broadly referred to as peripheral blood biomarkers, offer a noninvasive window into tumor biology. Based on this principle, the concept of liquid biopsy has emerged and gained momentum in both research and clinical settings. While various body fluids can serve as substrates for liquid biopsy, peripheral blood is the most accessible and has been studied the most [6]. Key blood‐based biomarkers include circulating tumor cells (CTCs), circulating tumor DNA (ctDNA), EVs, cell‐free RNAs (cfRNAs), peripheral blood mononuclear cells (PBMCs), and proteins. Each biomarker type contributes unique and complementary information. Advancements in detection technologies have significantly improved the sensitivity, specificity, and throughput of biomarker analyses, generating complex, high‐dimensional datasets. However, extracting actionable insights from such datasets poses significant analytical challenges. In this context, artificial intelligence (AI) has emerged as a powerful tool, capable of integrating multimodal data, uncovering hidden patterns, and therefore facilitating the discovery and validation of predictive biomarkers [7]. AI‐based models can enhance risk stratification, predict treatment response, and support clinical decision‐making with unprecedented precision [8]. The development of these novel technologies has led to specialized differentiation within the field and an expanded range of available biomarkers. To select the most suitable biomarkers and detection strategies for specific clinical needs, it is necessary to stay informed about the latest technologies for these biomarkers and their applications.

In this review, we systemically examine the current landscape of peripheral blood biomarkers in solid tumors, focusing on their biological underpinnings, detection platforms, and clinical applications in diagnosis, prognosis, and treatment monitoring. A comparative analysis of biomarkers was then conducted, alongside an exploration of advances in integration strategies based on the complementarity among biomarkers. Despite ongoing challenges in the clinical translation of circulating biomarkers, they hold significant promise for improving patient outcomes.

## 2 Overview of Major Peripheral Blood Biomarker Classes

Peripheral blood has emerged as a rich source for biomarker discovery, expanding new dimensions in tumor diagnosis and treatment. The major biomarker classes are either products shed from tumor lesions or key mediators in the dynamic evolution of tumor and tumor microenvironment (TME). Each of them has its own origins and functions, which can be captured and analyzed by corresponding detection technologies. These provide noninvasive insights into tumor biology, progression, and TME dynamics with a distinct focus. CTCs demonstrate a clonal relationship with the originating tumor, allowing for multiomic profiling and functional assays. And ctDNA carries tumor‐specific genetic alterations such as mutations, copy number variations, or methylation. Their direct link to the tumor can mirror the tumor's genetic aberrations at both cellular and molecular levels. EVs and cfRNA exhibit greater heterogeneity in their origins that are not limited to tumor cells, thus bridging tumor cells and the TME. EVs secreted by different cells encompass different cargoes to transmit signals during intercellular communication during tumor progression or treatment, and cfRNA captures transcriptional changes in tumor and TME cells during this process. In the context of host–tumor interactions, PBMCs and proteins serve as key mediators in immunological and functional profiling. Collectively, these biomarkers provide comprehensive information on the natural and treatment‐induced evolution of the tumor.

## 3 Circulating Tumor Cells

CTCs are malignant cells that detach from primary or metastatic lesions and enter the peripheral blood or lymphatic system. First described by the Australian physician Thomas Ashworth in 1869, CTCs have since emerged as a pivotal biomarker for solid tumors in peripheral blood. Although a unified theory describing the complete phylogeny of CTCs remains elusive, it is unequivocal that the presence of CTCs suggests the potential for tumorigenesis and metastasis, and key steps in the metastatic cascade involving CTCs have been delineated [9, 10]. In the process of detachment and intravasation, most CTCs are rapidly cleared from the circulation through apoptosis induced by shear stress or immune surveillance mechanisms such as phagocytosis. However, an intelligent subset of CTCs that survives this hostile environment was observed to undergo dynamic phenotypic and molecular adaptations, including alterations in genomic, transcriptomic, and epigenetic landscapes [11], and some of them even exhibit characteristics of cancer stem cells [12, 13]. They were also observed to form clusters that included not only individual CTCs but also other cells, such as platelets and neutrophils, which shield CTCs from immune attack and facilitate extravasation [9, 14]. Moreover, CTCs possess tumor‐related temporal dynamics, with fluctuations in their abundance associated with circadian rhythms and intermittent dormancy. The presence of these heterogeneous characteristics within the CTCs population poses challenges for CTCs detection while also presenting new opportunities for CTCs utilization in solid tumors.

### 3.1 Detection Technologies for CTCs

Current technologies for the enrichment, identification, and downstream analysis of CTCs are undergoing continuous refinement to address the limitations imposed by their scarcity and high heterogeneity in their clinical applications. Meanwhile, it is also imperative to meticulously plan the preanalytical steps preceding sample processing, including the anatomical site and time of blood draw, the type of blood collection tube utilized, the volume of blood collected, and the conditions of transportation and storage [15]. These factors exert a substantial influence on the consistent detection and interpretation of CTC data, and guidelines for their standardization are under investigation to improve their clinical utility potential.

**FIGURE 2:**
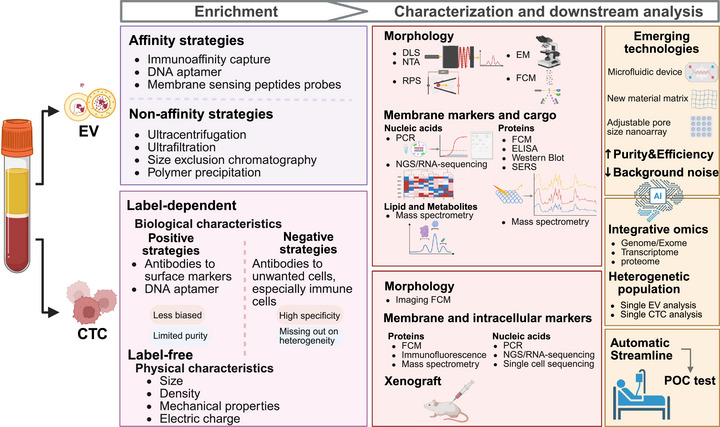
Detection technologies for CTC and EV. EV, extracellular vesicle; CTC, circulating tumor cell; DLS, dynamic light scattering; NTA, nanoparticle tracking analysis; RPS, resistive pulse sensing; EM, electron microscopy; FCM, flow cytometry; PCR, polymerase chain reaction; NGS, next‐generation sequencing; ELISA, enzyme‐linked immunosorbent assay; SERS, surface‐enhanced Raman spectroscopy; POC, point‐of‐care; AI, artificial intelligence. Created in https://BioRender.com.

As the first and most critical step in the entire process of CTCs analysis, isolation and enrichment refer to the process of separating CTCs from blood cells and other components based on their unique biological or physical properties, including both label‐free and label‐dependent enrichment strategies [16, 17] (Figure 2). The former rely on physical characteristics of CTCs, including size, density, mechanical properties, and electrical charge, which enable a less biased capture but with limited purity. The latter can be further divided into positive and negative strategies based on different target cells. The positive strategy harnesses antibodies or various aptamer probes attached to microfluidic or magnetic devices to identify specific surface markers of CTCs, thus has higher specificity but greater risk of missing out heterogeneity. The negative enrichment strategy is similar to label‐free methods, but it has the opposite goal of depleting unwanted cells [10]. It has also been acknowledged as a method for unbiased selection of CTC phenotypes, but concerns regarding yield and purity persist. In addition to identifying more biomarkers on CTCs [18], an increasing number of novel devices and materials, such as microfluidic devices and aptamer probes, are being employed to improve the sensitivity and specificity of CTCs enrichment. The following table provides some illustrative examples (Table 1). It is notable that the selection of an appropriate strategy and technology should be contingent upon the purpose of the application and the requirements for subsequent identification and analysis.

**TABLE 1:** Advancement in the technology of CTCs’ enrichment.

Methodology	Technology	Improvement	References
Label free	Multistage microfluidic chip compromises size‐based microcolumn and stiffness‐based cone channel	Improve the recovery efficiency and the purity of CTCs mixed by leukocytes by 1.8‐fold	[19]
	Microfluidic acoustophoresis chip	Detect larger number of phenotypic CTCs and heterogenetic CTC clusters with higher sensitivity	[20]
Label dependent (positive)	Microfluidic channel with a scalable microscale mesh and nanofunctionalized micropores	Address the issue of flow rate limiting capture efficiency in surface‐capture device	[21]
	Magnetic particles with nanofilopodia	Enable higher efficiency and specificity for CTCs detection than smooth particles	[22]
	Magnetic particles modified by dual aptamers	Capture CTCs expressing different surface biomarkers simultaneously	[23]
	Lubricant‐infused surface grafted with recognition peptides	Effectively reduce nonspecific adhesion of untargeted cells and blood components	[24]
	Aptamer linked to DNAzyme via DNA framework	DNAzyme‐catalyzed proximal protein biotinylation increase capture sites to identify CTCs with low biomarker expression	[25]
Label dependent (negative)	High‐throughput microfluidic chip with force‐amplifying magnetic lenses	Process larger sample volumes and enhance the efficiency of leukocytes depletion with lower clogging risk	[26]

Subsequent to the enrichment process, it is necessary to identify CTCs using a series of features, including morphology, specifically expressed proteins detected by immunofluorescence, and genomic variant traits detected by polymerase chain reaction (PCR) or sequencing. AI shows great potential for feature mining (Figure 2). AI‐assisted image analysis has been integrated into stain‐free identification of CTCs. In a proof‐of‐concept experiment, it succeeded in distinguishing more than 97% of CTCs and discriminating between two types of CTCs from neuroblastoma and ovarian cancer with an accuracy of over 97% [27]. An unsupervised deep transfer‐learning algorithm called CTC‐Tracer was introduced to transfer histogenesis information from primary tumor cells to CTCs via their single‐cell RNA sequencing (scRNA‐seq) profiles, thereby identifying CTCs from background cells and revealing their origins as well as gene markers [28].

### 3.2 Clinical Application of CTCs

#### 3.2.1 Screening and Diagnosis

The detection of CTCs facilitates the clinical diagnosis of tumors in the gray zone of traditional tumor markers, and their synergistic effects in predictive models developed using machine learning methods support higher diagnostic accuracy. In a clinical study, the number of CTCs exhibited the capacity to distinguish between prostate cancer patients, those with benign prostatic hyperplasia, and healthy donors, with no statistically significant correlation in prostate‐specific antigen (PSA)  levels among the three groups [22]. Then, the PSA level, free to total PSA value, and CTCs number were integrated by support vector machine to obtain a model with high sensitivity (100.0%) and an area under the curve (AUC) value (98.1%) [22]. In another study, CTCs were extracted from both portal venous and peripheral blood samples and combined with CA19‐9 expression levels to construct predictive models using a neural network framework, leading to a substantial increase in the average accuracy for CA19‐9‐negative pancreatic patients from 47.1 to 87.1% [29]. Additionally, the synergy of aptamer‐based probes, enzyme‐free nucleic acid amplification, and metal nanoparticles enables the precise and quick identification of CTCs [30]. The double‐stranded DNA with an aptamer first specifically binds proteins on CTCs (e.g., HER2, MUC1) and then releases single‐stranded DNA, which triggers catalyzed hairpin assembly amplification and frees the metal nanoparticles, and finally initiates cascading signal amplification through selective cation exchange reaction or other interaction to obtain the quantitative information of CTCs [31, 32]. This strategy has been proven to yield consistent results comparable to those of imaging and pathological tests, and hopefully push forward the utilization of portable devices, such as hand‐held fluorometers, for point‐of‐care (POC) tests of CTCs [30], which removes obstacles to the translation of their clinical diagnostic value.

#### 3.2.2 Prognosis Monitoring

The association between the presence and enumeration of CTCs and the risk of reduced overall survival (OS) and metastasis has been extensively studied. In patients with pancreatic ductal adenocarcinoma (PDAC), the presence of CTCs has been shown to have a marked negative prognostic impact on OS, as evidenced by a significant reduction in OS from 28.6 to 8.5 months [33]. Negative correlation between CTCs counting and OS has also been reported for patients with locally advanced or metastatic bladder cancer or prostate cancer [34, 35]. They further performed immunofluorescent staining and whole transcriptome sequencing to analyze protein and messenger RNA (mRNA) expression in CTCs, respectively, which revealed invasive protein markers in the CTCs subpopulation and identified distinct RNA expression profiles from patients with varying degrees of tumor burden [34]. In another study, RNA sequencing was performed on 273 CTC samples from 117 patients with metastatic prostate cancer, yielding four transcriptional phenotypes that are consistent with lineage states identified in tissue, which are predictive of progression and survival [36]. Therefore, a more comprehensive characterization of CTCs beyond mere enumeration could significantly expand their clinical utility. Moreover, it has been reviewed elsewhere that the clinical value of dynamic CTCs detection for monitoring minimal residual disease (MRD) in patients with various malignant tumors [37]. In a prospective study, perioperative and follow‐up CTC levels were evaluated and successfully predicted recurrence within two years after surgery, with an AUC of 0.9786, earlier than radiological recurrence with a median lead time of 183 days [38]. In practical clinical applications, the combination of CTCs and cell‐free DNA (cfDNA) can effectively enhance the sensitivity of MRD detection, and further characterization of these markers could provide insights into the molecular evolution of MRD to guide disease management [39].

#### 3.2.3 Therapy Response Predicting and Target Discovery

It has been demonstrated that the initial or ongoing burden of CTCs can mirror and assess the patients’ responses to therapy, indicating the value of guidance in clinical trials of medications [40]. Patients with Stage II colorectal cancer (CRC) would benefit from adjuvant chemotherapy (ACT) when preoperative CTCs were ≥4 per 7.5 mL of blood, and receive no benefit when preoperative CTCs were <4 [41]. CTCs have been observed to decrease or convert to negative with effective chemotherapy or radiotherapy in numerous cancers, which is used to predict relapse or response [42]. The qualitative and quantitative detection of programmed cell death ligand 1 (PD‐L1) expression on CTCs fluctuates during immune checkpoint inhibitor (ICI) therapy, which facilitates the selection of patients eligible for ICI and is expected to serve as an alternative to tumor tissue PD‐L1 testing [43]. However, the more definitive conclusions await validation in larger sample sizes and extended follow‐up durations [43]. Furthermore, CTCs carry markers of drug resistance, which can inform the adjustment of treatment regimens. A recent biomarker study of the PRESIDE Phase b trial has reported new evidence that patients with metastatic castration‐resistant prostate cancer benefit from continuing enzalutamide with docetaxel when the presence of CTCs expressing androgen receptor splice variant 7 is absent [44]. Nevertheless, the clinical significance of early identification of nonresponders by CTC detection and subsequent switch regimens to improve clinical outcomes remains to be confirmed [45]. The advent of single‐cell sequencing and the construction of organoids from CTCs signifies a pioneering advancement in the downstream analysis of CTCs (Figure 2). These methodologies provide a more comprehensive characterization of primary and metastatic tumor cells, thereby helping to determine potential treatment targets and devise new potential treatment strategies, which is an essential trend in the clinical application of CTCs within the paradigm of precision medicine [46, 47]. In a recent study of organoids derived from metastatic breast cancer CTCs, a method for the long‐term expansion of the organoids was established based on the identification of the neuregulin 1–HER3 axis [48], which promotes the growth of CTCs and may become a promising therapeutic target.

### 3.3 Summary

CTCs represent a dynamic and heterogeneous population of cells that provide critical insights into metastasis biology. Their rarity in blood has led to rapid advances in detection technologies and emphasized the necessity of meticulous consideration of enrichment strategies. The downstream analysis of CTCs is closely correlated with clinical applications, where the potential of feature‐rich CTCs has gone beyond simple enumeration as they contain comprehensive information about the primary tumor and metastasis that can be used not only for multiomics analysis but also for establishing cell lines, organoids, or patient‐derived xenograft models for in‐depth study. The challenges persist due to technical heterogeneity and the absence of standard operating procedures.

## 4 Circulating Tumor DNA

cfDNA is fragments of DNA released into the bloodstream during cellular processes, including cell death (apoptosis, necrosis), inflammation, and nuclease activity [49]. The release of cfDNA can occur through both passive and active mechanisms [50], resulting in heterogeneous patterns of cfDNA. They display different fragment lengths and structures, such as nucleosomal protein‐associated, transcription factor binding site‐associated, and vesical‐associated cfDNA [49]. ctDNA is a subset of cfDNA that originates from tumor cells, including CTCs, and TME. Therefore, ctDNA should have contained the same genetic characteristics as their origin cells [51], which can be used to differentiate ctDNA from cfDNA of nontumor origin [52] then calculate the ratio named tumor fraction. It has been found that the abundance of ctDNA correlates with tumor burden, and comprehensive genomic profiling of ctDNA shows high concordance with matched tumor tissue, thereby better recapitulating tumor heterogeneity and identifying additional actionable genomic alterations for therapy [53]. However, discrepancies between ctDNA and tissue‐derived mutation profiles have also been observed [54], which highlights the underexplored areas of tumor molecular features associated with ctDNA release [50]. Variability in ctDNA shedding across individuals and tumor types introduces uncertainty into clinical interpretation, where further mechanistic insights are needed. In addition to genetic alterations, nongenetic features of ctDNA—such as DNA methylation patterns (methylomics), fragment size distributions (fragmentomics), and nucleosome positioning (nucleosomics)—have emerged as powerful complementary biomarkers [52]. These features often appear more abundantly and consistently than somatic mutations, thereby can also predict tumor fraction [49] and may enhance the sensitivity and specificity of ctDNA‐based assays [55]. Continued exploration of both genetic and nongenetic features of cfDNA is expected to enhance its clinical relevance as a biomarker and drive its integration into clinical practice.

### 4.1 Detection Technologies for ctDNAs

**FIGURE 3:**
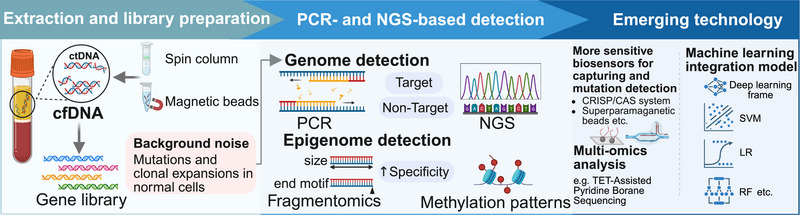
Detection technologies for ctDNA. cfDNA, cell‐free DNA; ctDNA, circulating tumor DNA; PCR, polymerase chain reaction; NGS, next‐generation sequencing; SVM, support vector machine; RF, random forest; LR, logistic regression. Created in https://BioRender.com.

Similar to those in CTCs, low analyte abundance in blood and preanalytical factors pose challenges to ctDNA analysis and necessitate rigorous quality control and standardization measures [15]. Plasma samples are a recommended source of ctDNA to minimize the impact of hemolysis and white blood cell autolysis on downstream analysis [51]. But the magnitude of this effect is still under investigation [56]. Efforts are being made to obtain a higher yield of ctDNA by optimizing protocols for sample preparation and storage and developing tailor‐made extraction kits. Besides, larger plasma volumes may also help if permitted [57]. At present, techniques for quantifying and characterizing ctDNA can be broadly classified based on whether they involve PCR or next generation sequencing (NGS) (Figure 3). They can also be classified based on the genomic coverage of ctDNA components in blood, which is divided into targeted and untargeted methods [58]. The main challenges arise from the large amount of background noise caused by the widespread accumulation of somatic mutations and clonal expansions in normal human tissues [59]. New amplification strategies are continually being developed, utilizing ever‐improving capture materials and sensors to achieve higher sensitivities. For instance, superparamagnetic beads or nanoparticles have been functionalized as surfaces for capturing ctDNA and amplifying signal [60, 61]. Several electrochemical sensors utilize the clustered regularly interspaced short palindromic repeats (CRISPR)‐associated nuclease system to achieve ultrasensitive and reliable detection of mutant ctDNA [62, 63, 64]. In addition to improving somatic mutation detection, analysis of methylation and fragmentomes offers a mutation‐agnostic, tumor‐naïve ctDNA approach that may address the challenges [65, 66]. Bisulfite sequencing has been a standard method for measuring DNA methylation, but it is limited by the requirement for large amounts of input DNA when using exponential amplification. The newly developed assay, linear amplification‐based bisulfite sequencing, allowed the detection of underrepresented DNA components with a lower input amount [67]. Cell‐free methylated DNA immunoprecipitation and sequencing is another method that has been used to analyze both symmetrically methylated and hemi‐methylated regions to obtain additional biomarkers [68]. Moreover, TET (ten‐eleven translocation) enzyme‐assisted pyridine borane sequencing can exclusively convert methylated cytosines, thus showing less destructive effects than bisulfite sequencing and opening the possibility of simultaneous methylome and genome analysis [69]. In the future, the definitive trend in ctDNA detection will be a more straightforward sequencing method that can simultaneously capture multiomics information, including genome and epigenome data, combined with multimodal analysis.

Therefore, traditional analytical approaches have been insufficient to address the increasing complexity and volume of ctDNA‐related data (Figure 3). Currently, machine learning and advanced computational algorithms are being increasingly employed to integrate and interpret high‐dimensional ctDNA datasets, enabling the discovery of novel biomarkers and improving predictive performance [70, 71]. An algorithm named MisMatchFinder has successfully inferred mutational signatures of ctDNA across multiple cancer types when using shallow whole‐genome sequencing data and constructed accurate predictive models [24]. A stacked ensemble model integrating five different cfDNA fragmentomic features demonstrated superior sensitivity for early detection of lung cancer [25]. cfMethylPre, a novel deep learning framework, has been developed to handle the high dimensionality of cfDNA methylation data. It pretrained a large language model on DNA sequence information and integrated it with DNA methylation profiles, which enhances the model's ability to capture complex genomic patterns and identify key cancer‐associated genes [72].

### 4.2 Clinical Application of ctDNA

#### 4.2.1 Screening and Diagnosis

Compared with tissue biopsy, ctDNA detection offers a minimally invasive, reproducible, and time‐efficient method that improves the efficiency of early cancer screening. The level of ctDNA can be used to assess the need for follow‐up tissue diagnosis before starting therapy [73]. In cancers of unknown primary, the characterization of cfDNA, such as methylation profiles [74], repeat sequences, and fragmentation [75], can be a valuable addition to histopathological analysis to trace the tissue of origin and address uncertainty about diagnosis, prognosis, and therapy strategies. These multidimensional characterizations are also used to build diagnostic models that distinguish early‐stage cancers in a more cost‐effective way. In a prospective cohort study, four types of cfDNA characteristics, including fragment size pattern, copy number variation, nucleosome coverage pattern, and single nucleotide substitution, were profiled to detect gastric cancer signals and then accurately classified patients and noncancer individuals in an ensemble model [76]. Differentially methylated regions between tumor and nontumor samples provide tumor‐specific ctDNA methylation biomarkers [77]. Researchers identified the top 500 methylome‐wide CpG sites that differ between epithelial ovarian cancer and healthy women and built an early diagnostic model with 80% sensitivity and 95% specificity [78]. Another multicenter cohort study found that the methylation‐based ctDNA model outperformed the mutation model in detecting hepatocellular carcinoma (HCC), and researchers subsequently selected two methylation markers using machine learning algorithms to train robust diagnostic models [79]. Furthermore, subtype‐specific patterns of DNA methylation can be detected in ctDNA and have been reported to establish subtype classifiers that may guide precision therapy [80]. In cases of benign and malignant lesions that are challenging to discern on imaging, cfDNA has become a useful diagnostic tool for more precise noninvasive classification [81]. Researchers further integrated the cfDNA‐based model and the radiomic model to reduce misdiagnosis rates and optimize treatment decision‐making [82]. Although these studies faced limitations such as relatively small cohort sizes, they offered promising methods for tumor screening.

#### 4.2.2 Prognosis Monitoring

Due to the short half‐life of ctDNA, it may be released from CTCs and micrometastasis after surgical resection of the primary tumor or neoadjuvant therapy [83]. Therefore, a considerable number of clinical trials have been conducted or are currently underway to detect MRD by ctDNA analysis in patients with various types of solid tumors, especially CRC, intending to predict clinical outcomes such as progression‐free survival (PFS and OS [83]. Studies have revealed that the detectable ctDNA, both at baseline and following treatment modalities, is significantly associated with worse OS and elevated risk of relapse [84, 85]. They have also found that a lead time exists between ctDNA‐based MRD detection and imaging‐based detection of recurrence, which varies by tumor type and stage [86, 87]. In a large cohort with a median follow‐up of 23 months from the CIRCULATE‐Japan GALAXY prospective observational study, ctDNA‐based detection of MRD is predictive of OS and disease‐free survival in patients with resected CRC, and ctDNA positivity was associated with poor OS and higher mortality [88]. It has been observed that the mean cfDNA concentration of patients with advanced tumors is significantly higher than that of those with earlier stage disease [84], and the addition of ctDNA quantification at diagnosis to established risk factors may improve the prognostic stratification of patients [86, 89].

Additionally, longitudinal measurements of the genomic landscape in ctDNA can capture intra‐tumor heterogeneity and track tumor evolution, which serve as indicators of phylogenetic patterns correlated with metastatic dissemination and poor clinical outcomes [90, 91]. In a large retrospective cohort study of patients with advanced or recurrent endometrial cancer, those with TP53 mutations or other somatic alterations detected in ctDNA had an inferior OS [92]. Novel detection technologies and bioinformatic tools have been developed to track tumor mutations in cfDNA and extract clonal composition, thereby offering insights into the clonal structure of residual lesions and the genesis of metastatic disease [93]. Meanwhile, promoter hypomethylation and enrichment of stemness‐associated transcription factors in cfDNA of metastatic castration‐resistant prostate cancer patients have been detected, which may provide a mechanistic explanation for the disease's lethality [94]. Detection of ctDNA by tumor methylation‐specific assay has also been shown to predict relapse or progression following macroscopically complete surgery [95]. Preliminary studies have identified the quantification of ctDNA in specific cancer types or the number of ctDNA mutations independent of cancer type, potentially correlated with a higher rate of venous thromboembolism, but external validity necessitates further investigation [96, 97]. Finally, for neoplasms causally linked to viral infection, such as Epstein–Barr virus (EBV) positive nasopharyngeal carcinoma, viral cfDNA profiles can dynamically inform real‐time recurrence risk during treatment [98, 99].

#### 4.2.3 Therapy Response Predicting and Target Discovery

Changes in ctDNA occur at diverse time points during treatment and disclose distinct patterns depending on the treatment modality, which correlate with clinical response variables [100]. The concentration of cfDNA is observed to decrease in patients with a good response to combined ICI and stereotactic ablative radiotherapy, which could provide biomarkers for improved immunotherapy regimens [101]. Compared with single‐tissue biopsies, ctDNA analysis can offer a more comprehensive assessment of tumor mutational burden (TMB) and microsatellite instability, which are linked to therapeutic responses [102]. When evaluating efficacious treatment options for extensive‐stage small cell lung cancer, low on‐treatment TMB, as indicated by ctDNA, was associated with longer progression‐free survival and OS [103]. Moreover, the patient‐specific somatic tumor mutations in cfDNA also allow close monitoring of the allele fractions of tumor variants in plasma, which hold predictive value in determining treatment responses [104]. Therefore, ctDNA status, including its genomic and epigenomic features, yields novel insights for stratification strategies to identify populations that would benefit from different therapeutic regimens, especially ACT, facilitating the precise treatment decisions on therapy escalation and de‐escalation [45]. The Phase 2 DYNAMIC trial is the first to use ctDNA to guide adjuvant therapy and found that those with negative postoperative ctDNA can achieve similar recurrence‐free survival (RFS) after receiving de‐escalated chemotherapy as those receiving standard chemotherapy, which may allow omission of ACT in ctDNA‐identified low‐risk patients without compromising RFS [105]. And ctDNA‐positive patients showed an OS benefit after ACT in a Phase 3 trial [106]. Longitudinal monitoring of ctDNA clearance in these patients can inform treatment efficacy, with higher ctDNA clearance suggesting superior OS [88, 106]. Another study established a blood‐based genomic immune subtypes scheme that integrates the features inferred from ctDNA, including TMB, chromosomal instability, and intra‐tumor heterogeneity, to determine whether immunotherapy should be added to first‐line chemotherapy in non‐small cell lung cancer (NSCLC) patients [107]. The ctDNA genotyping is also applied to match patients with advanced cancer to more efficient targeted therapies [108]. For specific mutations associated with response or resistance to targeted therapies, ctDNA has been shown to function as a detectable carrier and thus guide treatment in patients with these mutations [109, 110, 111]. Furthermore, multiple plasma‐based assays have successfully detected novel actionable alterations in ctDNA beyond known mutations, which have the potential to supplement or supplant tissue biopsies in exploring new therapeutic targets and elucidating mechanisms of resistance [112]. A workflow of extended combined genomic profiling based on cfDNA, tumor, and germline analysis has been proposed to improve the detection yield of actionable variants. It can broaden therapeutic options and reveal genetic predispositions that may relate to treatment efficacy [113].

### 4.3 Summary

Under pathological conditions, the release and clearance dynamics of cfDNA, including ctDNA, undergo continuous changes, and the underlying biological mechanisms still require further investigation. They carry distinct mutations and variations, and the selection of detection methodology depends on the study design. Methylation has emerged as a promising field, offering higher sensitivity and specificity, as well as enhanced capability of tissue‐of‐origin identification. As ctDNA testing expands to broader clinical indications, well‐designed prospective trials are essential to establish its clinical utility, cost effectiveness, and role in personalized cancer management.

## 5 Extracellular Vesicle

EVs are lipid bilayer‐enclosed structures secreted by a wide range of cell types into the extracellular space. In line with the recommendations from the *Minimal Information for Studies of EVs 2023* (*MISEV2023*), the term “EVs” is preferred when specific subtypes cannot be clearly distinguished due to limitations in isolation and characterization methodologies [114]. Classifications based on cellular origin, physical properties, or molecular composition should be applied with caution, as certain isolation techniques may copurify heterogeneous populations or fail to resolve their precise subcellular origins [114]. Size‐based distinctions are also commonly used, with 200 nm often serving as the threshold between small and large EVs. Functionally, EVs serve as versatile carriers of diverse biological cargos—such as nucleic acids, proteins, and metabolites—that reflect the physiological state and functional dynamics of their parent cells. These cargos are protected from degradation within the vesicular structure, making EVs pivotal mediators of intercellular and cell–extracellular matrix communication [115]. Due to their ability to modulate various biological processes, EVs have garnered considerable interest as potential biomarkers for both physiological and pathological states. Multiple databases have been developed to aggregate EV‐related data from published studies, supporting the growing body of evidence for their diagnostic and prognostic utility [115]. In the context of cancer, EVs play a crucial role in tumor progression, metastasis, and remodeling of the TME. They act as conduits for crosstalk between malignant and nonmalignant cells, influencing immune responses and contributing to therapeutic resistance, and are thus positioned as promising targets for cancer diagnosis and treatment [116, 117]. Emerging studies have demonstrated that EV‐encapsulated biomolecules—including DNA [118], noncoding RNAs (ncRNAs) [119], proteins [120], and metabolites [121]—possess significant diagnostic and therapeutic value, owing to their stability and disease specificity. Currently, the direction of effort for the precise detection of EVs is the multiomics profiling at the level of single EVs [122], and the application of AI in interpreting complex data to realize more efficient biomarker screening is a pivotal driver for the future of EV research.

### 5.1 Detection Technologies for EVs

The International Society of Extracellular Vesicles has discussed prevailing approaches to EV enrichment and characterization and provided recommendations to assist researchers in determining purity and identifying EVs [114]. However, besides the presence of contaminants with overlapping size and density, the intrinsic heterogeneity of EV subpopulations and that arising from different host factors and sample preparation conditions pose significant challenges to EV isolation and downstream analysis [123, 124]. Consequently, the two main goals of technological advancement are to achieve the efficient isolation and purification of EVs from complex clinical samples and to enable highly sensitive and specific analysis of heterogeneous EV populations [125].

The existing isolation techniques primarily include ultracentrifugation, size‐based methods such as ultrafiltration and size‐exclusion chromatography (SEC), polymer precipitation, and immunoaffinity capture (Figure 2). The principles, as well as advantages and disadvantages of these techniques, have been comprehensively reviewed elsewhere [120, 126]. Meanwhile, enhanced and novel technologies in this field are constantly developed. In size‐based methods, the application of new materials as SEC matrix enables the production of EVs that are almost free of soluble proteins and lipoproteins [127]. A nanoarray with adjustable pore size not only allows size‐dependent isolation but also acts as a nanoreactor for further characterization [128]. Immunoaffinity capture combined with a plasmonic gold nanowell structure has successfully amplified signals of single EVs that may be missed by traditional fluorescence imaging [129]. Other affinity methods independent of antibody, such as targeted nanoarray modified by specific aptamers [130] and nanotrap‐structured microparticles [131], can also detect biomarkers on tumor EVs even with very low expression levels and purify EVs in a damage‐free way. Unlike antibody‐mediated capturing, membrane‐sensing peptide probes capture EVs based on their membrane physical traits rather than specific protein epitopes that may be complementary to antibodies [132]. A two‐pronged strategy has been developed that first immobilizes EVs by specific antibodies and subsequently detects them by DNA aptamers, which ensures the removal of most non‐EV substances and enables the subsequent quantification of biomarkers on EVs of different tumor assignments [133]. Moreover, a hook‐like structure was designed onto the pH‐low insertion peptide to prevent dissociation from the TME–EV membranes during a pH change from the TME to circulation, and biotinylation enabled the subsequent magnetic harvest of TME‐specific EVs [134]. Microfluidics‐based techniques are increasingly available to meet the requirements for higher yield, purity, and efficiency. They are integrated with traditional isolation methods that rely on the physical property or immunoaffinity to achieve more rapid capture and a lower limit of detection (LOD). For instance, a microfluidic chip was designed to fill with microbeads that can generate a nonuniform electric field and utilize dielectrophoresis to capture EVs, which achieved a LOD of 161 particles/µL180; when filling with antibody‐conjugated microbeads, the LOD further improved to 54 particles/µL with specific capture [135]. Additionally, the microfluidic devices utilize different microchannels, such as spiral or irregular serpentine configurations, to further enhance impurity removal [136, 137].

Next to the enrichment process is the assessment of the purity, physicochemical, and biochemical characteristics of EV samples (Figure 2). MISEV2023 has listed and offered guidelines for mainstream methods for estimating EV abundance based on specific numbers, particle size, and quantification of protein and/or lipid content, as well as technologies for characterizing EV morphology, protein components, and nonprotein markers [114]. Due to the heterogeneity of EVs, there is no single molecular measurement that can quantify all EVs, and no universal molecular markers of EVs or EV subtypes [138]. In contrast, surface‐enhanced Raman spectroscopy (SERS), as a label‐free sensitive technique, places more emphasis on analyzing the entire EV system and providing cancer‐type‐specific SERS signals [139]. A proof‐of‐concept study has demonstrated that the combined use of tumor‐specific Raman tags and spectral signals from label‐free regions enhances interpretability and improves classification accuracy [140]. It is recommended to introduce multiple measures of EV concentration and to implement complementary methodologies to characterize EVs adequately [114, 138]. In order to quantify specific EVs, novel materials are utilized to develop sensors that detect single‐digit levels of EV particles at the nanoliter sample level, such as a microstructured optical fibre sensor [141]. Nanocomposites have been reported in a sensor that allows multimodal detection of EVs, including temperature, absorbance, and fluorescence intensity [142]. DNA walker, a kind of artificial nanomachine, is also a promising biosensing strategy for EVs detection [143]. Additionally, chips for integrating isolation and analysis, or a one‐step strategy bypassing the need for isolation, are preferred. A microfluidic dielectrophoresis enzyme‐linked immunosorbent assays (ELISA) chip with a colorimetric sensor has been designed that is capable of electrically capturing EVs and detecting biomarkers in them [144]. Another microfluidic chip induces fusion of EVs with charged liposomes that load molecular beacons to detect target mutations within mRNA or microRNA (miRNA) contained in EVs, eliminating the need for prior EV isolation or RNA preparation [145]. These novel detection strategies offer a streamlined workflow, effectively reducing noise from non‐EV or nontumor signals [146, 147]. In the future, it is a key trend to automate composite detection devices to improve testing consistency, which holds promise as a valuable tool for POC test and expands the clinical application of EVs [148] (Figure 2). POC test‐oriented detection using handheld devices is under intense investigation, where aptamer‐driven rolling‐circle amplification reactions and photothermal transducers for signal conversion are usually adopted [149, 150]. AI algorithms have demonstrated significant advantages in the processing and integration of complex spectrum and image data from EVs and in the establishment of classifiers or predictive models for cancer screening [151].

### 5.2 Clinical Application of EVs

#### 5.2.1 Screening and Diagnosis

The number and components of EVs, especially those derived from tumors, would change depending on tumor type, disease stage, or response to therapy, which provides a rich source of biomarkers for detecting cancers, including screening asymptomatic populations and confirming diagnosis [152]. The majority of studies have focused on the inclusion of previously characterized biomarkers within EVs and the identification of novel ones that are differentially expressed in tumors, and these biomarkers have been tested in clinical trials for the early detection of various cancer types [116, 152]. Nanoflow cytometry assays have confirmed differential concentrations or counts of specific EVs of tissue‐ or tumor‐derived origin in the presence of tumors, such as EVs expressing CD63 and CD81 in glioblastoma [153] and EVs carrying thyroid transcription factor‐1 and surfactant protein B in NSCLC [154], which demonstrates exciting screening potential superior to existing imaging examinations. Aptamers targeting a predictor of CRC metastasis were fluorescence modified and used to simultaneously detect positive CTCs and EVs for more precise diagnosis [155]. Although no EV protein markers are currently approved for diagnostic use, EV proteins have been the most widely explored as potential biomarkers through various analytical methods and patient cohorts [156]. Multiplex profiling of EVs’ membrane epitopes has identified a group of surface proteins that are overexpressed in cancer patients compared with healthy controls [157]. A DNA aptamer‐encoded strategy with an AND‐logic gate can be used to combine multiple membrane protein markers, yielding more accurate diagnostic signals [158]. In the early phase of new, unknown EV protein biomarker discovery, untargeted proteomic analysis performed by mass spectrometry (MS) remains the prevailing approach [156]. A great number of extensive EV proteomics studies have been conducted across a range of patients with specific cancer types or subtypes and healthy controls to identify crucial proteomic signatures for diagnosis and stratification [159, 160]. In‐depth analysis of these data using machine learning helps extract the most valuable features and build robust models [161, 162]. Protein is not the only component differentially contained within EVs. There is ample evidence to support the use of other components as cancer‐specific biomarkers. The diagnostic value of EV ncRNAs in early detection of multiple cancer types has been reviewed in a recent study [163]. The deconvolution method can distinguish cancer cell‐derived EVs from healthy cell‐derived EVs based on miRNA‐seq data [164], and cancer cell‐derived EV RNA can be detected as transcripts of tumor‐associated mutations for the following diagnosis [165]. The distinct enrichment profiles of EV RNAs in patients with malignant, precancerous, or benign lesions have been revealed and used to train diagnostic models [166]. There is also increasing interest in exploiting the aberrant glycan patterns on tumor‐derived EVs as reliable biomarkers for diagnosis [167, 168]. Innovations and advances in systems or sensors that employ SERS allow sensitive, holistic profiling of molecules in EVs to differentiate between cancer and noncancer origins [169, 170]. Additionally, multidimensional data on EVs, such as proteomic characteristics and size heterogeneity, incorporated by AI algorithms can be leveraged for multicancer detection [171].

#### 5.2.2 Prognosis Monitoring

Tumor‐derived EVs act as a bridge for intracellular communication in the TME, profoundly impacting diverse facets of tumor progression, such as invasive and metastatic activity [172]. For example, N2 tumor‐associated neutrophils, derived from polarized neutrophils in the TME, produce EVs that transfer miR‐4745‐5p/3911 to gastric cancer cells, downregulating the gene expression of slit guidance ligand 2 and ultimately promoting metastasis [173]. Molecular profiling of EVs has revealed characteristics distinct from those of the primary tumor, suggesting the presence of metastatic lesions [174]. A panel of four miRNAs within EVs has been identified as biomarkers for lymph node metastasis among patients with CRC [175]. To assess the diversity of EV phenotypes, Cieslik et al. performed a numerical analysis of fluorescence signals from EVs with various physical properties, finding that increased diversity was significantly associated with shorter survival [176]. A prospective cohort study detected concentrations of both CTCs and PD‐L1^+^EVs and found that the combined analysis could assist in identifying patients with worse OS [177]. In aggregate, the potential of EVs as prognostic biomarkers for cancer management warrants further validation in larger populations.

#### 5.2.3 Therapy Response Predicting and Target Discovery

EV cargos correlate with changes in tumor behavior during treatment, thus have been evaluated to assess the effectiveness of therapies and resistance development [178]. The presence and modification of PD‐L1 on EVs have been used to predict response and resistance to immune therapy as a surrogate for PD‐L1 tissue proportion score (TPS) assessment. Schöne et al. reported that a high baseline level of PD‐L1^+^ EVs in blood was associated with better response to immunotherapy and prolonged survival, especially in patients with low or absent PD‐L1 tissue expression [179]. And the tumor PD‐L1 TPS can be assessed by the phosphorylation level of EV PD‐L1 [180]. Researchers have found that the EBV‐encoded BRRF2 is secreted via EVs and can disrupt signaling pathways for innate immunity. The association between increased levels of BRRF2^+^EVs and diminished response to immunotherapy in nasopharyngeal carcinoma patients was then observed, offering a potential biomarker for immunotherapy resistance [181]. B7‐H3 is another immune‐regulatory ligand that can also be detected on EVs, and the level of B7‐H3^+^ EVs increases when patients with prostate cancer develop resistance to hormone therapy [182]. Moreover, ncRNAs contained in tumor‐derived EVs have been demonstrated to mediate intracellular crosstalk to regulate immune responses, which can guide multiple strategies targeting ncRNAs to interfere with tumor progression [183]. In summary, EVs provide real‐time insights into tumor behavior that may affect treatment response, thereby helping improve and personalize therapeutic strategies.

### 5.3 Summary

EVs are a vast and heterogeneous group of cell‐derived membranous structures that transport various biomolecules for intercellular communication and thus play a crucial role in physiological and pathological processes. Confronted with this population, efforts focus on the emerging technologies such as personalized multiomics profiling, single EVs analysis, and AI‐driven analysis to facilitate more precise and portable detection technologies, which, when coupled with validation through standardized guidelines and large‐scale cohort studies, are accelerating the clinical translation of EVs.

## 6 Cell‐Free RNAs

cfRNAs circulating in the bloodstream originate either from the active secretion by viable cells or the passive release during cellular processes such as necrosis and apoptosis, including those occurring in malignant cells. To date, a wide array of cfRNAs has been identified and extensively investigated as promising biomarkers across various cancer types. These cfRNAs encompass both mRNAs and a broad spectrum of ncRNAs. Beyond transfer RNAs and ribosomal RNAs, ncRNAs can be further categorized based on their size and functional attributes into long ncRNAs (lncRNAs) and small ncRNAs, such as miRNAs, PIWI‐interacting RNAs, and small interfering RNAs [184]. cfRNAs are often encapsulated within EVs or associated with RNA‐binding proteins, forming stable ribonucleoprotein complexes [184, 185], which effectively shield them from RNase‐mediated degradation. Analysis of cfRNA fragment lengths has revealed that a substantial proportion remains intact and unfragmented in circulation [186]. This characteristic endows cfRNAs with enhanced stability and detectability in blood specimens, particularly during the early stages of tumorigenesis. As an integral part of the broader tumor‐derived blood biomarker landscape, cfRNAs can provide unique insights into transcriptomic alterations, including pathogenic alternative splicing events and gene fusions [187], which provides a new perspective for monitoring tumors through blood.

### 6.1 Detection Technologies for cfRNAs

The discovery process for cfRNA markers typically encompasses the following steps: blood isolation, RNA isolation, library preparation and sequencing, and bioinformatic analysis. These steps involve various factors that can introduce variations in results and existing studies have sought to shed light on potential obstacles at each step and to propose solutions [187]. At present, consensus is lacking on the optimal protocol for cfRNAs detection, given that the selection of different methodologies for isolation at the outset can exert a significant effect on downstream processes [185]. Fortunately, some applications have been created. For instance, based on comparative studies of 10 miRNA isolation methods, researchers developed an interactive web‐based application named miRDaR. It can return a ranked list of these methods according to their complexity, reproducibility, and threshold miRNA level, enabling the customized selection of methods [185]. Currently, most cfRNA isolation kits recover only a fraction of circulating cfDNA and carry an inherent risk of DNA contamination. Spectrophotometry is a common technique employed to assess the quantity of cfRNA after its extraction from blood samples. Subsequently, high‐throughput technologies, primarily including droplet digital PCR, quantitative reverse transcription PCR (RT‐qPCR), microarrays, and NGS, were used to analyze the gene expression characteristics of cfRNA [188]. In order to achieve more robust and precise quantification of cfRNAs, various detection strategies based on CRISPR methods have been reported, enabling the analysis of low‐abundance cfRNAs with high efficiency [189, 190, 191, 192]. Recently, Chen et al. developed a type of hydrogel microbeads that realized extraction‐free detection of miRNAs, aiming to minimize loss and degradation during RNA extraction procedures [193]. They successfully quantified three specific miRNAs for lung cancer and established a predictive model for subtype classification with the aid of machine learning [193]. As research into miRNAs as potential disease biomarkers progresses, interest is growing in mRNAs and lncRNAs that exhibit tissue‐ or disease‐specificity [194]. In several studies, the utilization of profiling data from cancer cell lines and tumor tissue, along with the analysis of multiple tissue datasets, has led to the identification of potential biomarker targets, which have subsequently been validated in blood cohorts [195, 196]. In other studies, owing to reductions in sequencing costs and technological advancements, diverse protocols for library construction that are not encumbered by limitations in fragment structural and input plasma volume have been developed to discover a broader range of cfRNAs species [194, 197]. The processed raw data were then utilized to identify potential biomarkers via comparative analysis of cfRNA profiles or machine learning methods.

### 6.2 Clinical Application of cfRNAs

#### 6.2.1 Screening and Diagnosis

Aberrantly expressed genes in tumor tissue generate distinct levels of transcripts, which are anticipated to become diagnostic biomarker candidates in circulation. Albrecht et al. applied unsupervised data mining to compare the transcriptome in melanoma and nontumor skin.They identified four differentially expressed genes and  their cfRNAs demonstrate high diagnostic accuracy [196]. Similarly, Metzenmacher et al. found high expression of MORF4L2 in NSCLC tumor tissue, and its cfRNA was significantly abundant in plasma samples from patients [198]. Additionally, plasma total RNA sequencing enables the detection of disease‐relevant cfRNA biomarkers. In CRC, three phases of discovery, total RNA sequencing, targeted capture sequencing, and RT‐qPCR for validation were performed to explore potential cfRNA biomarkers [199]. Meanwhile, microbiome‐derived plasma cfRNAs were also identified in RNA‐seq, and modifications in them can reflect the intrinsic status and activity of the microbiota in response to the tumor, making them more sensitive for detecting early‐stage cancer [200]. These validated cfRNAs can serve as diagnostic signatures to train machine learning models [201]. Another area of interest is the composition and cellular origin of cfRNA in the blood, as well as the alterations to its transcriptomic landscape, which offer a glimpse into tumorigenesis and progression. Liver‐targeted cellular deconvolution revealed changes in liver cell‐type proportions, thereby improving the performance of the diagnostic gene marker model [202]. Nullomers are the shortest sequences absent from the human genome and are related to tumor development. It has been reported that the number of emerged nullomers is correlated with the number of mutations across patients with cancer [203]. Recent findings indicate that cfRNA can serve as a carrier for those identified nullomers, facilitating the detection of cancer [203].

#### 6.2.2 Prognosis Monitoring

Dynamic alterations in cfRNA are indicative of tumor load and heterogeneity. Distinct variations were reported in the plasma transcriptomic profile among the various melanoma patient groups with different tumor burden or brain metastases, which reflected different states of tumors and helped identify the existence of metastases [204]. In breast cancer, the level of miRNA‐200 family in circulation has been reported to correlate with the formation of metastasis lesions [205]. Baseline levels and posttreatment changes of cfRNA have been shown to be valuable for prognostic prediction. A prospective study examined levels of circulating miRNAs in blood specimens collected from CRC patients who underwent surgical resection at four time points (preoperation, one week days, one month, and six months postoperation). The study found that persistently high levels of miRNA‐21‐5p after surgery were suggestive of tumor recurrence and progression [206]. cfRNA has also been utilized to predict MRD and the risk of postoperative recurrence. Modlin et al. developed a probabilistic model to assess the risk of prostate cancer based on blood mRNA signature. When applied to patients undergoing radical prostatectomy, it could distinguish patients by scoring: those with complete tumor removal (R0 and no biochemical or image confirmation of residual or recurrent disease) had low risk scores, and others showed no decline in risk scores [207]. Furthermore, it was determined that postoperative cfRNA markers provide an earlier warning of recurrence when combined with ctDNA [208], suggesting the potential benefit from integrating analysis to improve the sensitivity of MRD detection.

#### 6.2.3 Therapy Response Predicting and Target Discovery

Correlations have been identified between fluctuations of cfRNA and the patient's response to treatment. A study found that the elevated levels of five miRNAs, miR‐223‐3p, miR‐20a‐5p, miR‐17‐5p, miR‐19a‐3p, and miR‐7‐5p, were reduced in CRC patients responding to three months of 5‐Fluorouracil‐based therapy and rebound increase after six months, which may suggest the conversion of treatment from responsive to resistant phase [209]. In another study, the high level of MORF4L2 cfRNA in NSCLC patients decreased in those who achieved partial remission to treatment, when the levels of two classical tumor markers decreased in all patients [198]. Besides, cfRNA can detect therapeutic targets in NSLCLC tissue, such as oncogenic fusions and splicing variants, so as to obviate the need for laborious tissue biopsies [210]. Similarly, various alterations of the androgen receptor can be detected in cfRNA, which is associated with the rise of resistance to androgen receptor‐directed therapy in prostate cancer and thus has the potential to guide patient management [195]. Furthermore, cfRNA and their interacting genes or proteins participate in the process of drug resistance formation, and may therefore constitute innovative therapeutic targets in the future with the elucidation of underlying mechanisms [211]. In a Phase 2 clinical trial of single‐agent regorafenib in chemo‐refractory metastatic CRC, high MIR652‐3p expression was more common in patients who experienced progression as best response than in those who achieved partial response or stable disease. They further performed functional analysis of patient‐derived organoid models and pointed out that overexpression of MIR652‐3p was exclusively triggered by regorafenib and could overcome regorafenib resistance by impairing regorafenib‐induced lethal autophagy and orchestrating a switch from neoangiogenesis to vessel cooption. Notably, there was no prognostic effect of MIR652‐3p overexpression independent from regorafenib treatment, suggesting that MIR652‐3p expression might serve as a specific biomarker of resistance to regorafenib rather than a simple prognosticator [212].

### 6.3 Summary

cfRNA is derived directly from cellular transcription processes, so its expression levels can reflect real‐time cellular functional status, such as abnormal tumor cell proliferation or immune cell activation. With advances in technology, low‐abundance but large‐species cfRNAs are being explored and may become novel biomarkers. In the future, more convenient detection methods for POC tests need to be developed, and larger‐sample, longitudinal studies may help realize the clinical value of cfRNA.

## 7 Peripheral Blood Mononuclear Cells

The immune system plays a pivotal role in the initiation, progression, and therapeutic response of solid tumors. While prior studies have primarily concentrated on tumor‐infiltrating immune cells—key components of the TME [213]—emerging evidence indicates that PBMCs in cancer patients also undergo profound quantitative and functional alterations, which may have significant diagnostic and therapeutic implications [214]. PBMCs are a heterogeneous population of mononuclear cells circulating in peripheral blood, originating from hematopoietic stem cells in the bone marrow. They are mainly composed of lymphocytes, monocytes, and dendritic cells. In the context of solid tumors and related treatments, PBMCs exhibit dynamic changes and can traffic to various tissues and organs via the circulatory system. Notably, there is bidirectional regulation between PBMCs and TME that indicates heterogeneity in treatment response, where the transcriptional state of TME influences the immune compartment in peripheral blood, and these immune cells will enter the TME to perform their duty [215]. Compared with cfDNA and CTCs, PBMCs are more abundant and stable in peripheral blood, facilitating easier processing, storage, and retrospective analysis. These advantages position PBMCs as a promising complementary biomarker to existing tools, potentially offering more profound insights into the systemic immune dysregulation associated with tumor presence and progression.

### 7.1 Detection Technologies for PBMCs

Generally, PBMCs can be isolated from peripheral blood by density gradient centrifugation, and their immunophenotype is detected by flow cytometry. The past few decades have witnessed a series of key advances in flow cytometry technology, which can be extended to classical staining designs with additional lasers. However, this multicolor flow cytometry technique also has some limitations, including autofluorescence and fluorescence compensation issues. Cytometry by time‐of‐flight (CyTOF) is another high‐throughput detection technology that avoids the compensation problem of traditional fluorescent labeling. However, the information on cell size and intracellular complexity is lost, and it has a slower collection speed, higher sample preparation requirements, and a more limited number of markers compared with traditional flow cytometry [216]. Recently, the incorporation of machine learning techniques has enhanced the understanding of the systemic immune profile in cancer patients, leading to the development of various screening and diagnostic models. Based on information of different populations and the activation states of white blood cells, Dyikanov et al. developed a platform based on multidimensional machine learning to identify patients from healthy donors and categorize them into five immunotypes [217]. Similarly, Zhang et al. applied CyTOF to profile the composition and number of immune cells altered in patients with malignancies, which provided a foundation for constructing multicancer screening models using a random forest (RF) algorithm [214]. Additionally, fluorescent imaging or PCR, combined with machine learning and AI, offers novel opportunities to detect extensive chromatin remodeling and DNA methylation changes during PBMC activation [218, 219]. With the addition of new analysis methods, these traditional technologies for PBMCs can better reveal the systematic immunological variation and its associations with clinical behaviors, making them practical for prospective validation and eventual clinical deployment.

### 7.2 Clinical Application of PBMCs

#### 7.2.1 Screening and Diagnosis

There is increasing evidence of tumor‐associated alterations in PBMCs, which may become a novel biomarker for early cancer diagnosis [220]. A salient change involves the metabolic pathways in immune cells in response to tumor‐associated antigenic stimuli [221]. The diagnosis model developed from metabolic activity profiles of immune cells showed superior accuracy in detecting lung cancer at early stages, suggesting that immune activation can be monitored before other circulating biomarkers appear in peripheral blood when the tumor reaches a certain size [221]. Additionally, a cross‐sectional study compared mitochondrial functions in PBMCs and ovarian tissues from patients with epithelial ovarian cancer and healthy controls. It not only demonstrated mitochondrial disorders but also the consistency of mitochondrial changes in PBMCs and tumor tissues [222]. However, its dynamic process needs further exploration in larger, longitudinal cohorts. Moreover, systemic alterations in the tumor‐driven immune system can also be reflected in the transcriptomes of PBMCs. Martinez‐Usatorre et al. performed whole‐transcriptome analysis of PBMCs to identify overlooked biomarkers involved in CRC progression, paving the way for the development of peripheral blood transcriptomic analysis for early cancer screening [223]. Furthermore, given that tissue‐specific expression can be inferred from blood transcriptomes to predict certain disease states, it is hypothesized that PBMC transcriptomes contain information about the immune state in the TME [224]. Cao et al. predicted the major immune cell types and their expression levels in the TME using PBMC scRNA‐seq and identified a new immune signature of the TME inferred from blood that was associated with response to ICB treatment [225].

Similar to CTCs and cfDNAs, PBMCs can offer tumor‐specific DNA methylation changes. As PBMCs may mimic the bio‐status of tissues in contact with them by altering gene expression and epigenetic profiles, some researchers have examined whether changes in gene methylation levels in tumor tissue are reflected in PBMCs. Genome‐wide methylation of PBMCs is adopted to reveal the DNA methylation landscape and explore new differentially methylated positions. They found that the level of global gene methylation in PBMCs was significantly lower in patients, similar to the change in DNA methylation in local cancer tissue, and may be related to the immune system activation in the early stage of the tumor [226]. This result indicates that the methylation status of peripheral blood leukocytes effectively predicts tumor presence, which provides additional evidence for the application of PBMC methylation in early cancer diagnosis. Wang et al. developed a multiplex quantitative methylation‐specific PCR (BC‐mqmsPCR) assay based on four breast cancer‐specific methylation markers in PBMCs with an AUC of 0.936 and sensitivity of 90.0% [218]. It is noted that models combining traditional tumor markers with these epigenetic biomarkers have better diagnostic performance than those using traditional tumor markers along [218, 226]. Therefore, PBMCs likely play complementary roles to CTCs and cfDNAs, given their advantages in extraction and preservation.

#### 7.2.2 Prognosis Monitoring

Due to immune system changes during tumor development, detecting it is of great significance for assessing prognosis and predicting disease progression. Lymphocyte subsets are biomarkers of immune function and are closely related to the tumor prognosis. In various solid tumors, CD4^+^T cell lymphopenia has become a predictive factor of poor clinicopathological features and prognosis [227]. Previous studies have indicated an immune senescent or exhausted subset characterized by CD28^−^ and CD8^+^ surface markers was negatively correlated with tumor immunity and clinical outcomes [228]. However, dynamic alterations in peripheral CD28^−^CD8^+^T cells were observed in a retrospective cohort of patients with untreated HER2‐positive metastatic breast cancer, and those with high or increasing levels of CD28^−^CD8^+^T cells at baseline or during treatment had better tumor immunity and benefited more from HER2‐targeting therapy [229]. Therefore, the roles of T cell subsets need further exploration in larger, more comprehensive cohorts to assess their complex effects on tumor burden and prognosis. In addition to flow cytometry, DNA methylation‐based immune phenotyping, combined with deconvolution algorithms, has been developed to provide information across different subtypes of immune cells in peripheral blood [230, 231]. Applying the 12 immune‐cell‐type DNA reference matrix, Luo et al. distinguished alterations in immune cell type composition across different diseases, including cancers [232]. A study on bladder cancer showed associations between elevated neutrophil, basophil, and regulatory T cell counts, decreased CD4^+^ T memory cells, and an increased risk of death and recurrence [233]. They further divided patients into five groups with distinct immune and DNA methylation profiles identified by integrating algorithm and deep learning models, which broadens the scope of the effects of peripheral immune profiles on cancer prognosis [233]. Due to the high cell specificity and measurement accuracy of DNA methylation, cytometry based on DNA methylation may become more efficient than flow cytometry for immune profiling.

#### 7.2.3 Therapy Response Predicting and Target Discovery

The growing prevalence of immunotherapy is driving significant changes in cancer management and complicating clinical decision‐making. So it further emphasizes the growing need to learn more about immune‐related biomarkers that can help patients choose immunotherapy regimens. The final goal is to optimize and personalize treatment while reducing unnecessary costs and avoiding potential immune‐related adverse events (irAEs) in patients unlikely to benefit from treatment. T cells activate and differentiate into various functional subtypes under the guidance of specific cytokines, which not only participate in antitumor immunity but also tumor immune escape or immunosuppression, making them a key target of immunotherapy [234]. Before receiving ICB, baseline levels of naïve T cells (T_N_) and memory T cells (T_M_) in peripheral blood are positively correlated with treatment efficacy, while the levels of senescent subsets are negatively correlated with treatment prognosis. Studies have shown that in advanced NSCLC, the absolute count of CD4^+^T_N_ cells is an independent protective factor [235] and the proportion of CD28^−^CD57^+^CD8^+^T cells (senescence‐type cells) predicts an unsatisfactory response to ICI [236]. Additionally, the expression level or ratio of PD‐1 on circulating CD4^+^ T cells and CD8^+^ T cells at baseline is consistent with that in the tumor, indicating PD‐1/PD‐L1 axis activation status and thus ICI sensitivity. Cohort studies have shown that an elevated CD8^+^PD‐1^+^ to CD4^+^PD‐1^+^ ratio indicates more effective ICI treatment [237]. The composition of the T cell population and PD‐1 expression levels will change throughout treatment, so dynamic monitoring is equally crucial for assessing treatment response and benefit. ICI treatment induces the activation and proliferation of T cell subclusters that express *HLA‐DRA*, *CD38*, and *KI67* [238]. It has been observed that the rising level of *KI67* expression in blood CD4^+^ T_M_ cells is associated with treatment outcomes [239]. Tada et al. reported that the proportion of CD4^+^ and CD8^+^ terminal effector memory T cells increased in disease‐controlled patients with HNSCC after nivolumab injection [240]. Machine learning methods can help to discover new expression markers. In NSCLC, combined with machine learning methods, CD33^+^ monocytes were identified as specific subsets predicting ICI responsiveness, particularly in responders who had an increasing frequency of CD33^+^ monocytes [241]. irAEs occur when ICIs activate the immune system, which will affect multiple organs and impair patients’ quality of life [242]. The level and functional status of T cell differentiation subsets can help monitor irAEs. Although a high circulating level of CD8^+^CD28^+^ T cells is associated with better clinical outcomes, Geng et al. found that an excessive baseline level of these cells (≥309/µL) indicated a higher risk of severe irAEs [243]. Recently, a roadmap has been proposed to predict severe irAEs based on the correlation between elevated abundance of activated CD4 effector memory T cells and diversity of T‐cell receptor (TCR) in pretreatment blood and the development of irAEs [244]. For patients who developed irAEs, TCR sequencing analyses were performed to reveal peripheral blood T‐cell repertoire reshaping, which occurred on average five months prior to the clinical diagnosis of an irAE [245].

### 7.3 Summary

PBMCs are the mobile sentinels that mediate and reflect tumor‐driven alteration of systemic immunity. Traditional detection techniques combined with machine learning algorithms can precisely extract the immunophenotypes, transcriptomes, and methylation profiles of PBMCs. The clinical applications of PBMCs are closely related to tumor immunotherapy.

## 8 Proteins

In contrast to nucleic acid‐based biomarkers, proteins are the primary executors of cellular functions and the direct targets of numerous cancer therapies, underscoring their pivotal roles in cancer‐related liquid biopsy. The initiation, progression, and metastasis of solid tumors are closely associated with the dysregulation of intracellular signaling pathways, within which dynamic changes in protein abundance and epigenetic modifications serve as key drivers of tumor biology [246, 247]. Meanwhile, the dynamic changes in infiltration status of immune cells and their interactions with tumor cells within the TME can generate and release soluble immune factors that participate in the regulation of antitumor immunity and may be associated with resistance to ICB therapy [248, 249]. Consequently, profiling protein alterations offers critical insights into tumor cell activation, the characteristics of the tumor‐associated microenvironment, therapeutic responses, and systemic physiological impact. Importantly, these protein‐level transformations occurring within tumor lesions can also be detected in plasma, with some alterations exhibiting specificity to particular cancer types. Therefore, the application of protein biomarkers—either alone or in combination with other molecular markers—holds substantial promise for elucidating tumorigenic mechanisms and advancing personalized clinical management strategies.

### 8.1 Detection Technologies for Proteins

For the detection of single or a few protein biomarkers in liquid biopsies, traditional immunological methods are usually performed, including ELISA and chemiluminescence immunoassays, with multiple kits or sensors available, which has been a standard methodology in clinical practice and research. Although improved materials and techniques have emerged to develop a series of immunosensors with increasing sensitivity for analyzing those classical protein markers detection [250, 251, 252, 253], their specificity as independent predictors for solid tumors remains limited. Taking alpha‐fetoprotein (AFP) as an example, it has been a traditional but controversial protein biomarker for HCC diagnosis and surveillance, due to its serendipitous discovery, unknown function, and lack of consensus on a necessary biological link to HCC [254]. Consequently, efforts are underway to improve its performance in terms of sensitivity and specificity by combining it with imaging or by applying it within several statistical models [254, 255]. In contrast, high‐throughput proteomics, which continues to witness technological and methodological breakthroughs, has achieved promising results in identifying novel biomarkers [256]. Integrating with bioinformatics and multiomics analyses, proteomics provides a comprehensive framework for disease diagnosis, prognosis, and treatment [257]. Different proteomics methods show their advantages for specific research objectives. Given its capacity for untargeted proteomic research, encompassing total proteins and their modified forms, MS is preferred during the initial stage of biomarker discovery [258]. Antibody arrays are utilized to compensate for the inability of MS to detect low‐abundance proteins, while antigen arrays are effective for investigating proteins and their interactions with other molecules, a notable example being the study of tumor‐associated autoantibody profiles [259, 260]. In the context of targeted proteomics, techniques that employ nucleic acid hybridization, such as aptamer‐based assays or proximity extension assays, offer benefits in minimizing batch effects and ensuring reproducibility [256]. Furthermore, the rise of AI and the advent of machine learning algorithms have not only addressed the limitations of conventional protein biomarkers, such as the XGBoost model for HCC [261], but also paved the way for leveraging proteomic data in conjunction with large amounts of clinical and health‐related data, thereby facilitating the detection of multiple cancers [262]. The majority of studies adhere to a systematic workflow that involves the discovery of candidate proteins, the network analysis or expression verification, and the development of models for validation [260].

### 8.2 Clinical Application of Proteins

#### 8.2.1 Screening and Diagnosis

Plasma protein biomarkers have demonstrated promise in early tumor detection. In the realm of currently available protein biomarkers for early tumor diagnosis, an increasing number of researchers are adopting combination strategies based on machine learning algorithms to develop sophisticated predictive models that overcome the limited predictive capacity of isolated biomarkers. Joshi et al. utilize multistage heterogeneous ensemble‐learning models, a machine learning strategy for handling complex data patterns, to identify breast cancer through multiple biomarkers (β‐hCG, PD‐L1, AFP) with superior accuracy [263]. Due to its high efficiency and low cost, achieved through advanced algorithms, it holds promise as a complementary approach to existing ones, such as ultrasound, mammography, or biopsy. It awaits validation in larger clinical trials [263]. Additionally, products of aberrant signaling pathways and gene expression in neoplasias that have been identified are detectable in the plasma and thereby serve as early diagnostic markers. Researchers found that the ITIH5 gene was specifically upregulated in cholangiocarcinoma using bulk RNA‐seq analysis. They subsequently validated elevated ITIH5 expression in tumor tissues and the serum, indicating the diagnostic value of serum ITIH5 [264]. The Mendelian randomization study also identified 13 plasma proteins that exhibited a significant association with the risk of CRC development, thus providing novel targets for screening initiatives [265]. Serum protein markers that reflect changes of the body's immune system and coagulation status induced by tumor proliferation and metabolism can also be used for the early detection of malignant lesions [266, 267]. Leveraging protein microarrays containing 1622 function proteins and machine learning techniques, Kajornsrichon et al. profiled autoantibodies in 26 serum samples from 16 intrahepatic CCA patients and 10 healthy individuals and identified a signature composed of three autoantibody biomarkers in conjunction with CA19‐9 for intrahepatic cholangiocarcinoma detection [259]. With regard to the exploration of more protein markers, researchers employed untargeted MS to quantify differentially expressed proteins, which were subsequently validated by targeted assays such as ELISA or western blot analysis. In the early diagnosis of PDAC, six proteins were found to be upregulated in the PDAC cohort, and two were subsequently validated by ELISA. The selected proteins demonstrated high diagnostic accuracy in PDAC patients at Stages I and II [268].

#### 8.2.2 Prognosis Monitoring

A considerable number of proteins present in plasma have been found to be tumor specific, which is associated with tumor progression and patient survival outcomes. A cross‐sectional study demonstrated that the classical diagnostic markers of HCC, AFP, and PIVKA‐II collectively predicted decreased OS and RFS and were associated with unfavorable outcomes after laparoscopic hepatectomy [269]. Additionally, in a large‐scale multicenter study, researchers validated AKR1B10, a secreted protein with superior diagnostic properties to AFP [270], exhibited remarkable proficiency in predicting the poor prognosis of HCC [271]. It is a dynamic process of tumor and the TME development; thus, continuous monitoring of plasma protein changes can effectively reflect tumor activity and estimate recurrence. The change rate of TRACP5b, a serum marker of bone resorption, was significantly higher in patients with giant cell tumor of bone with locally recurrent disease than in the nonprogressed group [272]. An et al. found that the longitudinal change of serum AFP in HCC patients receiving hepatic arterial infusion chemotherapy follows three distinct trajectories, which showed outstanding performance in predicting survival outcomes in posttreatment HCC [273]. With respect to immuno‐oncology markers, prospective cohort studies have established associations between pretreatment interleukin‐8 levels and worse OS in patients, with the incorporation of machine learning algorithms enhancing the robustness of the results [274]. Metastasis is a significant contributor to patient mortality, and its early prediction is imperative for improving disease management. Models combined with protein biomarkers have been devised to evaluate the risk of tumor lymph node metastasis [275]. It is anticipated that more prognostic predictive proteins with high sensitivity and specificity will be validated and applied in large‐scale cohort studies.

#### 8.2.3 Therapy Response Predicting and Target Discovery

Circulating protein biomarkers serve as a critical point of entry for predicting patient benefits from various treatments and adjusting treatment strategies. Several tumor‐associated serum markers used for diagnostic purposes have also demonstrated predictive and prognostic value in patients undergoing treatment for malignancies, but it is imperative to interpret their application in concert with other diagnostic examinations and clinical findings. The advent of proteomic technologies has enabled researchers to identify and quantify a broad spectrum of expressed proteins effectively. Guo et al. conducted MS analysis of plasma samples from esophageal adenocarcinoma patients before chemotherapy, and the patients were grouped according to tumor regression score, a measure of treatment response [276]. Then, differentially abundant proteins were identified between these groups, of which C1q complex and GSTP1 proteins were validated by ELISA and were expected to stratify esophageal adenocarcinoma patients before chemotherapy [276]. Similarly, Mondelo‐Macía et al. discovered 324 differentially expressed proteins between responder and nonresponder patients receiving immunotherapy, inspiring research into the biological functions of these proteins [277]. Researchers also performed comparative proteome analysis of tumor cell lines with sensitivity or resistance, then filtered potentially secreted proteins to select candidates. Serum concentrations of these selected proteins were subsequently assessed in patient samples and correlated with clinical and follow‐up data to determine their value in differentiating between drug‐resistant and nondrug‐resistant patients [278, 279]. Many of them may be functionally associated with patients’ baseline or acquired resistance, thereby revealing potential therapeutic targets and influencing the decision‐making regarding therapeutic interventions, especially the soluble immune factors. It has been observed that pretreatment plasma concentrations of soluble checkpoint molecules are related to exhaustion status of antitumor immunity, and the combination of sPD‐L1 and sCTLA‐4 can serve as a complementary predictive factor of response to PD‐1/PD‐L1 blockade therapy in patients with a high level of tumor tissue PD‐L1 expression [248]. Soluble Tim‐3 (sTim‐3), another promising soluble biomarker, is significantly elevated in the plasma of various tumors before treatment and associated with ICB efficiency [280, 281]. In two cohorts of metastatic clear cell renal cell carcinoma patients adopting different immunotherapy, compared with those with low sTim‐3, patients with high sTim‐3 have significantly reduced survival under nivolumab (anti‐PD‐1) monotherapy but have similar survival probabilities under nivolumab + ipilimumab (anti‐CTLA‐4), which may provide guidance for the selection of ICB strategy [280]. Vitro experiments had investigated the mechanism by which sTim‐3 promotes tumor progression and suppresses immunity [281]. They further found that blocking sTim‐3 production by the ADAM10 inhibitor could reverse these effects, indicating the potential of sTim‐3 as a therapeutic target for cancer treatment [281]. Furthermore, the heterogeneity of biomarkers across treatment stages means that protein markers identified in pretreatment samples lack the capacity to forecast how patients will respond over time to multiple courses of treatment. In light of the limitation, investigators have undertaken longitudinal cohort studies to explore the dynamics of proteins correlated with therapeutic response, aiming to enhance the accuracy of predictive models [282].

### 8.3 Summary

The field of circulating protein biomarkers represents an intersection of tradition and innovation. Traditional markers are revitalized when integrating with algorithms and optimizing models. Low‐abundance proteins or bulk‐differentially expressed proteins are detected by emerging technologies, which inspire further exploration of the biological functions that may shape the unique characteristics of tumors and TME. This, in turn, supports personalized therapy.

## 9 Integrated Analysis and Future Perspectives

### 9.1 Comparative Advantages and Limitations of Different Biomarkers

Different biomarkers offer distinct information dimensions and vary in sensitivity, specificity, and timeliness, as shown in the following table (Table 2). It suggests that there is no single biomarker that can fit all clinical demands, and that biomarker selection should depend on the specific status of tumors.

**TABLE 2:** Comparative advantages and limitations of different biomarkers.

	Sensitivity	Specificity	Timeliness	Information dimensions	References
CTC	Low (rare in peripheral blood of early‐stage tumors and larger volume blood is required)	High (tumor‐derived intact cells with specific genomic and phenotypic traits for versatile downstream analysis)	Moderate (short circulating time; handheld devices offer instant testing; time‐consuming downstream analysis)	Enumeration; single‐cell genomics; phenomics (EMT, target expression); organoid and xenograft model	[9, 30, 33, 48]
ctDNA	Low (low abundance and larger volume blood may help)	Moderate (tumor‐specific alterations, background noise from normal cfDNA or nontumor cells still exist)	High (advanced sequencing technologies and bioinformatics methods outperform in early cancer screening)	Tumor burden quantification; MRD; genomic alterations (mutations, CNVs); methylation patterns; fragmentomics	[15, 49, 53, 57, 283]
EVs	High (abundant and stable in circulation)	Moderate (released by almost all the cell types and hard to purify specific EV populations)	Moderate (captures real‐time cargo secretion; lacks standard preanalytical procedures; development of POC tests)	Phenotype characteristics; multiomics of molecular cargo (genomics, transcriptomics, proteomics)	[115, 138, 148, 152]
cfRNA	Moderate (more actively transported into circulation and more sensitive when analyzing functional pathways than ctDNA)	Moderate (host‐derived RNA background dominates, easy to be confounded by nondisease factors)	High (well‐established PCR, microarray and sequencing methods for quantification and qualification)	Quantitative changes; transcriptomics (alternative splicing, gene fusion); microbiome	[184, 232, 284, 285]
PBMC	High (abundant and stable in circulation)	Low‐moderate (immune dysregulation may overlap with nontumor conditions)	Moderate (isolated by density gradient centrifugation and characterized by flow cytometry or omics analysis)	Systemic immune status (genomic, transcriptomic, epigenetic changes during immune activation)	[216, 221, 223]
Protein	High (detectable in a very small amount of blood by MS or immunologic methods)	Low‐moderate (limited tumor specificity as the fluctuation in level influenced by various factors such as inflammation)	High (well‐established detection methods for fast testing and capturing dynamic changes)	Tumor‐specific proteins; proteomics; functional mediators (tumor‐associated autoantibody; soluble immune factors)	[258, 272, 273]

Abbreviations: CNV, copy number variations; EMT, epithelial‐mesenchymal transition; MRD, minimal residual disease; MS, mass spectrometry; POC, point‐of‐care.

### 9.2 Synergistic Applications of Multianalyte Liquid Biopsy

Given the complementarity of information dimensions provided by circulating biomarkers, synergistic analysis of multiple biomarkers is imperative for acquiring comprehensive, real‐time information on the natural and treatment‐induced evolution of tumors [286], where the application of AI in multidimensional predictive models holds great promise. First, for the individual biomarker, integrating different features can improve the performance of tumor detection. Given the potential interdependence between the fragment end motif and size of cfDNA, researchers introduced fragment end motif by size (FEMS) as a novel integrative feature [287]. The classifier based on FEMS outperformed standalone fragment end motif (AUC, 0.869) and fragment size (AUC, 0.780) classifiers, achieving an AUC of 0.917, and the AUC was further improved to 0.968 when combined with the classifier using genomic coverage features into an ensemble [287]. Second, when considering multiple biomarkers, combining tests leverage the advantages of each. It has been demonstrated that cfRNA analysis can potentially complement ctDNA analysis to broaden molecular characterization of tumors, as 11 of the 34 variants identified in tumor tissue genomic DNA were detectable in ctDNA and three in cfRNA [284]. Combined cell‐free multiomics analysis, including total and small cfRNA sequencing, cfDNA whole genome sequecing, and methylated cfDNA sequencing,  could substantially enhance the detection capacity for cancer genes, where the two analytes offered complementary genetic insights [288]. CTCs and ctDNA are additional partners with nonoverlapping detection profiles and complementary prognostic values [289]. Their synergistic effects have been explored that the combination of CTCs and ctDNA exhibited enhanced sensitivity for predicting recurrence compared with analyzing CTCs or ctDNA alone (85.7 vs. 75 or 70.4%), and double‐negative (CTCs–/ctDNA–) reached the best RFS time compared with those double‐positive or single‐positive patients [39]. The incorporation of ctDNA variant allele frequency at baseline (as a continuous variable) and CTC count (as a dichotomized variable) into multivariate clinicopathological models showed optimal prognostic efficacy [289]. With the assistance of machine learning, CTC enumeration was used to define a prognostic stratification model, and its integration with pathogenic mutations in ctDNA could predict treatment response and provide deeper insight into treatment resistance [290]. Additionally, the simultaneous detection of CTCs and EVs is possible by recognizing the same target molecules, which may enable more precise clinical diagnosis and prognosis [155, 177]. EVs could also combine with traditional protein biomarkers, such as PSA for prostate cancer. Researchers have found that low levels of prostate‐specific membrane antigen‐positive (PSMA^+^) EVs with low PSA levels could predict the most significant low risk of progression, which helps identify long‐term responders to treatment [291]. LIBERTYLUNG (NCT04790682) is an ongoing clinical trial designed to predict responses to first‐line immunotherapy in metastatic NSCLC by ctDNA together with circulating immune cells. In summary, the integration of multiclass biomarker data has the potential to overcome individual limitations, thereby improving understanding of tumor biology and predictive efficiency.

### 9.3 Current Challenges in Clinical Translation

Currently, a series of clinical trials has been conducted to explore the application of peripheral blood biomarkers (Table 3), demonstrating their significant potential for clinical translation, especially for treatment response and decision‐making. However, several bottlenecks remain before their full integration into clinical practice. Key challenges can be summarized in the following four aspects. The first is the lack of standardized protocols for the processing and detection of biomarkers with low abundance and inherent heterogeneity, especially for the CTCs [292] and EVs [293]. It is difficult to characterize rare biomarkers [44, 294], which gives rise to diverse detection methodologies that vary in sample collection, processing, and testing procedures, resulting in inconsistent results and affecting the statistical power to detect changes negatively [295]. Measures such as standardizing the timepoint of sampling and using larger volumes of blood could reduce error [26, 33]. Second, appropriate bioinformatics analysis is necessary for batch‐effect correction, distinguishing tumor‐derived signals from background noise and exploring the underlying biological mechanisms. Efforts have been made to optimize sequencing and bioinformatics analysis strategies to filter germline‐ and clonal hematopoiesis‐associated white blood cell variants in ctDNA detection [296] and platelet‐derived RNA contamination in cfRNA detection [284]. Third, larger‐scale, multicenter clinical trials with rigorous prospective study designs are essential for robust clinical validation, and the interpretation of these trials should be approached with caution, as the extent to which they improve clinical outcomes remains unknown. It is challenging to define appropriate control groups, select endpoints, and address bias. Many current trials are small or retrospective, impeding the generalizability of their findings [105, 295]. Meanwhile, studies with insufficient follow‐up time or focus on limited subtypes need further exploration of the exact effects of biomarkers. Finally, the detection assay can be costly, which will limit the accessibility and may potentially lead to overtesting and undue anxiety. Automated, streamlined assays have recently developed rapidly to achieve cost‐effective detection. As an emerging technology, relevant regulatory policies are still in a state of development, and the insurance policies also struggle to attain comprehensive reimbursement coverage [105].

**TABLE 3:** Summary of registered clinical trials investigating biomarkers for clinical application.

Trial identifier	Disease focus	Biomarker type	Key goals/outcomes	References
NCT04820868	Multicancer	ctDNA methylation	To construct a customized panel of 161 984 CpG sites for the early detection and localization of six types of cancers in the colorectum, esophagus, liver, lung, ovary, and pancreas. The multicancer detection model achieved high sensitivity, specificity, and accuracy in the real‐world simulation.	[283]
ChiCTR2400083525	Esophageal cancer	ctDNA methylation	To identify potential methylated DNA markers from differentially methylated esophageal cancer‐associated regions. A diagnostic model based on 3 of these markers was trained and validated in a multicenter clinical cohort with a sensitivity of 85.5% and a specificity of 95.3%.	[297]
ChiCTR2300072317	Early lung cancer	EV membrane proteins	To develop a diagnostic panel consisting of five EV membrane proteins (CD81, PDL1, GLIPR1, LBR, and SFTPA1) and validate its accuracy in distinguishing patients with early lung cancer from the control group in a validation cohort.	[298]
NCT06381583	EGA	microRNA	To identify six overexpressed miRNAs (miR‐106b, miR‐146a, miR‐15a, miR‐18a, miR‐21, and miR‐93) to establish a diagnostic signature and test its ability to screen esophageal adenocarcinoma and precancerous lesion.	[201]
NCT02974764	PDAC	Transitional CTCs	To assess the ability of consistent transitional CTCs to predict the risk of late recurrence in patients who were disease free at 1‐year postsurgery duration.	[295]
NCT01809691	Metastatic CSPC	CTCs count	To validate CTC count as a prognostic biomarker that estimates higher hazard of death and disease progression, and improves upon existing prognostic factors.	[299]
NCT01682083	Melanoma	ctDNA	To explore the prognostic value of baseline BRAF^V600^‐mutant ctDNA. It showed that patients with positive baseline ctDNA have significantly higher risks of recurrence and death, with ctDNA demonstrating greater predictive value for survival outcomes than biomarkers from tissue biopsy.	[300]
NCT01619111	HER2^−^ MBC	HER2^+^ CTCs	To compare effectiveness of standard therapy +/− lapatinib in HER2^−^ MBC patients with HER2^+^ CTCs. The clearance was significantly associated with improved overall survival, and these patients can benefit from HER2 targeted therapy.	[301]
NCT03147287	HR^+^/HER2^−^ MBC	CTCs enumeration	To identify two prognostic subgroups based on the baseline CTC enumeration in patients progressing on CDK4/6 inhibitors and identify patients that more likely to benefit from intensified therapies.	[302]
NCT03928210	MBC	CTC cluster size	To determine whether treatment with the Na^(+)^/K^(+)^ ATPase inhibitor digoxin could reduce mean CTC cluster size and explore the possible mechanism of dissolution, but clinical outcome endpoints were not assessed.	[294]
NCT03409848	Advanced HER2^+^ EGA	CTCs count HER2 expression on CTCs T cell repertoire richness	To identify patient subsets that could safely omit chemotherapy. It suggested that patients with favorable blood T cell metrics, absence of CTCs, or HER2 expression on CTCs equally benefit from only immunotherapy while patients without these markers need additional chemotherapy.	[292]
NCT03285321	NSCLC	ctDNA	To personalize the duration of consolidation immunotherapy treatment after chemoradiotherapy by detection of ctDNA before, during or after 6 months of the treatment, as the presence of ctDNA is associated with residual disease and inferior outcomes.	[303]
NCT03016312	Metastatic CRPC	ctDNA	To identify patients more likely to exhibit survival benefit from enzalutamide after abiraterone progression based on baseline and dynamics of ctDNA tumor fraction, which also provide complementary to traditional PSA testing.	[304]
ACTRN12615000381583	Colon cancer	ctDNA	To evaluate the long‐term efficacy of ctDNA‐guided adjuvant chemotherapy for Stage II colon cancer. It showed that the ctDNA‐guided approach could reduce the use of chemotherapy without compromising 5‐year recurrence‐free survival; and postoperative ctDNA burden and ctDNA clearance status at the end of treatment could further stratify the risk of ctDNA‐positive patients.	[105]
NCT03856411	NSCLC	ctDNA	To investigate the concordance between genomic sequencing of ctDNA and tumor tissue and confirm the independent predictive effect of ctDNA that exhibiting high TMB and mutations in the specific signaling pathways for patients benefited more from immuno‐chemotherapy.	[305]
NCT02680587	Oligometastatic CSPC	PSMA^+^ EV	To determine that low baseline concentrations of PSMA^+^EV are predictive of benefit from stereotactic ablative radiotherapy treatment.	[291]
NCT03010722	Metastatic CRC	microRNA	To find biomarkers of clinical benefit to the multikinase inhibitor regorafenib and identify MIR652‐3p as a biomarker and molecular driver of resistance to regorafenib.	[212]
NCT01485926	HER2^+^ breast cancer	PBMCs	To study the alterations in phenotype, genotype, and cytotoxic capacity of circulating immune cells in the context of response to chemotherapy in combination with trastuzumab, lapatinib, or both.	[306]
NCT03700476	Nasopharyngeal carcinoma	PBMCs	To identify a Ki67^(+)^ proliferating regulatory T cells population in PBMC samples from 12 pairs of matched relapsing and nonrelapsing patients following anti‐PD‐1 treatment, whose low baseline frequency could predict immunotherapy benefit.	[307]
NCT02134925	Colonic adenoma	PBMCs	To identified specific profile of prevaccination PBMCs in responders to vaccine therapy, where a significantly higher percentage of CD4^+^ naïve T cells and lower percentage of CD8^+^ T effector memory cells and CD16^+^ monocytes were observed.	[308]

Abbreviations: CRC, colorectal cancer; CRPC, castration‐resistant prostate cancer; CSPC, castration‐sensitive prostate cancer; EGA, esophagogastric adenocarcinoma; HR, hormone receptor; MBC, metastatic breast cancer; NSCLC, non‐small cell lung cancer; PDAC, pancreatic ductal adenocarcinoma; PMSA, prostate‐specific membrane antigen; TMB, tumor mutational burden.

### 9.4 Emerging Trends and Future Directions

The future of peripheral blood biomarker research will be driven by technological innovation, expanded applications, and improved clinical integration, with the increasingly widespread application of AI and machine learning methods set to play a crucial role [71]. In terms of detection technologies, AI has the capacity to perform extensive analysis of high‐dimensional data, thereby facilitating more precise identification of tumor cell characteristics and potential biomarkers closely associated with the occurrence and progression of malignant tumors. The following tables present recent examples of AI applications in biomarker detection (Table 4) and integrating models for clinical application (Table 5). Single‐cell sequencing enables profiling of cellular molecular heterogeneity and cell type annotation. The AI algorithm developed based on the approach can trace the original lesions of CTCs and enhance detection sensitivity [28]. Epigenetic analysis, such as DNA methylation, holds potential for multicancer screen when combined with machine learning models [283]. In terms of application, incorporating benign diseases into diagnostic models can expand the discriminatory efficacy of biomarkers across “malignant disease‐benign disease‐healthy”, thereby improving the clinical usefulness of models [82, 131, 309]. Biomarkers for MRD detection [4, 77] or for prediction of immunotherapy response [217, 241] would drive further exploration of the biological mechanisms that regulate tumor progression and guide subsequent clinical management. In the future landscape of clinical practice, the way forward lies in combination testing and portability device. As peripheral blood biomarkers cannot yet serve as surrogates for traditional tissue biopsy or imaging examination [310], it is critical to integrate these new assays with conventional methods to provide patients with more accurate and comprehensive information. In the future, the detection process will likely involve contributions from a variety of technology platforms, with the objective of detecting diverse types of markers and overcoming the problem of low early‐stage sensitivity while maintaining an acceptable level of specificity [311]. Additionally, the development of miniaturized devices for rapid, POC testing will reduce turnaround time from days to hours, enabling real‐time treatment adjustments.

**TABLE 4:** The application of AI in peripheral blood biomarkers detection.

Analytes	Detection technology	Main algorithm	Main results	References
CTC	scRNA‐seq	Domain adaptation learning	The CTC‐Traver algorithm was developed for CTC recognition, lesion tracing, and gene marker identification based on scRNA‐seq data of CTCs. The model demonstrated a prediction accuracy of >85% for the tissue origin of CTCs on eight independent datasets and could distinguish cancer types.	[28]
CTC	scRNA‐seq	RSF	The algorithm analyzed differentially expressed genes in CTCs and constructed bone metastasis‐related genes prognostic index based on the high dimensional gene data.	[312]
CTC	TPI‐FC	Hierarchical machine learning decision‐maker (shallow neural network)	The machine learning‐powered TPI‐FC distinguished tumor cells from white blood cells based on 3D morphological features and refractive index distribution with an accuracy of >97% and enabled the detection and typing of CTCs at single‐cell resolution without labeling.	[27]
CTC	RNA‐seq	RF, Gaussian finite mixture model	The model was used to evaluate the predictive capability of risk genes and finally selected four genes (TRIP10, NGFR, SLC48A1, and SRMS) to develop a risk score model for assessing the prognosis of colorectal cancer patients.	[313]
ctDNA	WGS	Two‐dimensional CNN, multilayer perceptron	A platform named MRD‐EDGE for detecting single nucleotide variant and copy number variant of ctDNA was designed. It improved signal‐to‐noise enrichment in WGS by ∼300× compared with previous WGS error suppression and reduced the degree of aneuploidy from 1 Gb to 200 Mb.	[314]
cfDNA	WGS	Penalized LR	The ARTEMIS (Analysis of RepeaT EleMents in dISease) approach was developed to detect repeated landscapes of cfDNA, which can identify the tissue of tumors and early detect cancer.	[75]
cfDNA	WGS	Fragle (a multistage machine learning model)	The model quantified ctDNA levels from a cfDNA fragment length density distribution, which can be used to explore correlation of ctDNA dynamics and treatment response, and detect MRD.	[315]
cfDNA	Targeted sequencing	RF	The model characterized and differentiated genetic signatures in cfDNA between gallbladder cancer and benign lesions and developed a cfDNA‐based model.	[82]
EV‐miRNA	TIRF	CNN	The deep learning model was used to automatically analyze TIRF images of single‐EV multi‐miRNAs and then classify different EV subpopulation. It identified the triple‐positive EV subpopulation that was the main variation EVs between 5 cancer cells and normal plasma.	[316]
EV	Interferometric plasmonic microscope	LDA	The algorithm was used to analyze the multidimensional matrix containing the correlative information between sizes and protein biomarkers of EVs and form the signature based on the correlation to determine cancer type with higher accuracy.	[171]
EV	SERS	Principal component analysis‐linear discriminant analysis	The machine learning algorithm‐powered SERS spectra analysis first successfully distinguished EVs derived from different cell lines (H8, HeLa, and MCF‐7 cell) and then the data were used to train a model that can identify serum EVs derived from cancer patients and healthy people.	[317]
cfRNA	RNA‐seq	CNN, LR	A novel method combining statistics and machine learning models called cfPeak was proposed, which can detect cfRNA peaks and efficiently mine low abundant cfRNA peaks with high importance as cancer markers.	[318]
EVs	Metabolomics	OPLS‐DA, LASSO	Screening for significantly different metabolites and identifying optimal marker combinations for entry into subsequent studies to differentiate between early gastric cancer, benign gastric disease, and healthy controls.	[131]
EVs	exoRNA‐seq	LR, LASSO, RF	A set of 12 exosomal tumor RNA signatures was confirmed as potential pan‐cancer biomarkers.	[293]
EVs	Proteomics	OPLS‐DA, RF, LASSO	The model screened for proteins significantly contributing to the differentiation of colorectal cancer from healthy controls and identified the most diagnostically valuable protein combinations‐PF4 and AACT.	[161]
NK cell	Fluorescence microscope	RF	The machine learning model was trained to extract and integrate multidimensional features of NK cell images, which can classify NK cell subpopulations with higher accuracy for further exploration of cancer immunotherapies.	[319]
Protein	OLINK proteomics PEA technology	RF, RSF	The algorithm identified six serum immuno‐oncology markers that were significantly associated with overall survival OSand/or PFSprogression‐free survival in metastatic breast cancer patients.	[274]
PBMC	Flow cytometry, RNA‐seq	Unsupervised clustering, elastic net regression	Five immunotypes with unique distributions of different cell types and gene expression profiles were identified and then used to generate immunotype signature scores, which can predict effectiveness of treatment.	[217]
PBMC	CyTOF	RF	The algorithm calculating the PBIScore according to the immune cell composition, phenotype, and function in peripheral blood, which translate the high‐dimensional characteristics into quantitative tumor diagnostic indicators.	[214]
PBMC	CyTOF	RF	The machine learning method analyzed high‐dimensional profile of PBMC in NSCLC and confirmed the predictive value of CD33^hi^ monocytes for anti‐PD‐1 effectiveness.	[241]

Abbreviations: RSF, random survival forest; CNN, convolutional neural network; LASSO, least absolute shrinkage and selection operator; LDA, linear discriminant analysis; LR, logistic regression; MRD, minimal residual disease; OPLS‐DA, orthogonal partial least‐squares discriminant analysis; RF, random forest; SERS, surface‐enhanced Raman spectroscopy; TIRF, total internal reflection fluorescence microscopy; TPI‐FC, tomographic phase imaging flow cytometry; WGS, whole genome sequencing.

**TABLE 5:** AI models of integrated biomarker analysis for cancer diagnosis, prognostic assessment, and treatment monitoring.

Analytes	Key biomarkers	Patient cohort/cancer type	Main algorithm	Main validation parameters of the model	References
Diagnosis					
CTCs	CTCs count, PSA, Free/Total‐PSA	Prostate cancer (*n* = 21); Benign prostatic hyperplasia (*n* = 15); HD (*n* = 10)	SVM	AUC, 0.981; Sn, 100%	[22]
cfDNA	Fragmentomics (CNV, fragment size distribution, nuclear features)	Training cohort (*n* = 133): 66 UCEC, 67 HD; Validation cohort (*n* = 89): 44 UCEC, 45 HD	Ensemble model of GLM, GBM, RF, DL, XGBoost	AUC, 0.994; Sn, 97.8%; Sp, 95.5%	[320]
cfDNA	Fragmentomics (CNV, fragment size distribution, fragment size ratio, nuclear features, mutational context)	Training cohort (*n* = 360): 176 CRC, 184 HD; Validation cohort (*n* = 236): 117 CRC, 119 HD; Prospective cohort (*n* = 242): 129 CRC, 113 HD	Ensemble stacked model based on GLM, GBM, RF, DL, XGBoost	Validation cohort: AUC, 0.986; Sn, 93.16%; Sp, 97.47%; accuracy, 95.34%; prospective cohort: AUC, 0.969; Sn, 91.47%; Sp, 95.58%; accuracy, 93.39%	[321]
cfDNA	Fragmentomics (end motif, fragment size, genomic coverage)	Korean discovery dataset: 218 lung cancer, 2559 controls; Korean validation dataset: 111 lung cancer, 1136 controls; Caucasian validation cohort: 50 lung cancer, 50 controls	Multimodal ensemble classifier based on CNN and multilayer perceptron	Korean cohort: AUC, 0.968; Sn, 95.5%; Caucasian cohort: AUC, 0.979; Sn, 94.0%	[287]
cfDNA	Fragmentomics (CNV, end motif, fragment size distribution, fragment size coverage, breakpoint motif)	Training dataset: 113 NSCLC, 113 HD; Validation dataset I: 81 NSCLC, 47 HD; Validation dataset II: 118 NSCLC, 70 HD; External validation dataset: 120 NSCLC, 120 HD	Stacked ensemble model based on GLM, GBM, RF, DL, XGBoost	Validation cohort I: AUC, 0.984; Sn, 91.4%; Sp, 95.7%; validation cohort II: AUC, 0.987; Sn, 84.7%; Sp, 98.6%; external validation cohort: AUC, 0.974; Sn, 92.5%; Sp, 94.2%	[322]
cfDNA	Differentially methylated regions, CNV, fragment size ratio	Discovery cohort (*n* = 300): 150 ESCC, 150 HD; External validation cohort (*n* = 60): 30 ESCC, 30 HD	RF, HMM, multidimensional scaling	AUC, 0.95; Sn, 87%; Sp, 95%	[323]
cfDNA	Differentially methylated regions, differentially hemi‐methylated regions	Training dataset (*n* = 215): 58 liver cancer, 77 brain cancer, 80 HD; Validation dataset (*n* = 56): 15 liver cancer, 20 brain cancer, 21 HD	Elastic‐net regularized GLM	Liver cancer: AUC, 0.990; Sn, 92.5%; Sp, 94.2%; accuracy, 93.3%; brain cancer: AUC, 0.983; Sn, 91.4%; Sp, 95.7%; accuracy, 93.0%	[68]
cfDNA	Differentially methylated regions	Training dataset (*n* = 1247): 503 epithelial ovarian cancer, 744 HD; Validation dataset (*n* = 625): 251 epithelial ovarian cancer, 374 HD	LASSO, MethylBERT (a model based on transfer learning)	LASSO‐EOC: AUC, 0.92; Sn, 83.67%; Sp, 89.04%; MethylBERT‐EOC: AUC, 0.97; Sn, 89.24%; Sp, 94.39%	[78]
cfDNA	cfDNA mutation signatures, radiomic features, CA19‐9, gender	141 gallbladder cancer, 62 gallbladder benign lesions (training:validation = 7:3)	Multimodal ensemble classifier based on cfDNA radiomic model	AUC, 0.97	[82]
Tumor‐derived EVs	EpCAM^+^PDL1^+^ EVs EpCAM^+^MUC1^+^ EVs PDL1^+^MUC1^+^ EVs	Training cohort (*n* = 120): 50 HCC, 50 cirrhosis, 20 HD; Validation cohort (*n* = 30): 10 HCC, 10 cirrhosis, 10 HD	LDA	HCC (AUC = 1.0), cirrhosis (AUC = 0.985), HD (AUC = 0.985); overall accuracy, 93.3%	[147]
EVs	4 EV‐circRNAs	Training cohort (*n* = 75): 25 HD, 25 Stage I–II GC, 25 Stage III–IV GC; Validation cohort (*n* = 30): 10 HD, 10 Stage I–II GC, 10 Stage III–IV GC	LDA	AUC, 92.5%; Sn, 86.7%; Sp, 90.0%; accuracy, 88%	[309]
EVs	11 metabolite biomarkers	Training dataset (*n* = 75): 25 GC, 25 benign gastric disease, 25 HD; Validation dataset (*n* = 75): 25 GC, 25 benign gastric disease, 25 HD	LDA	AUC, 0.95; Sn, 88%; Sp, 93%	[131]
EVs	EVs proteomic profile	109 CCA, 78 primary sclerosing cholangitis + HD (training:validation = 7:3)	Binary LR	AUC, 0.905; Sn, 75.8%; Sp, 87.5%	[324]
EVs	12 EV‐RNA signatures	818 patients of 8 cancer types, 194 HD (training:validation = 8:2)	RF	AUC, 0.983; accuracy, 0.848	[293]
EVs	5 EVs surface proteins	96 GC, 92 HD (training:validation = 8:2)	XGBoost	AUC, 0.9347	[325]
EVs	2 EV‐derived proteins, CEA, CA19‐9	Train set: 195 CRC, 47 benign colorectal diseases, 96 HD; Test set: 161 CRC, 55 benign colorectal diseases, 162 HD	RF	AUC, 0.963	[161]
ncRNA	6376 differentially expressed orphan ncRNAs	Training dataset (*n* = 840): 335 NSCLC, 505 HD; Validation dataset (*n* = 210): 84 NSCLC, 126 HD	Variational auto‐encoder, triplet margin loss	AUC, 0.971; Sn, 92.9%; Sp, 87.2%	[326]
miRNA	6 miRNA signatures	Training cohort: 96 esophageal adenocarcinoma, 64 HD; Testing cohort: 118 esophageal adenocarcinoma, 105 Barrett's esophagus or low‐grade dysplasia, 74 HD	XGBoost, AdaBoost	AUC, 91.9%; Sn, 82.5%	[201]
TCR	Cancer type specific TCR repertoire	Cancer cohort (*n* = 59); noncancer control cohort (*n* = 120)	CNN, long short‐term memory	AUC, 0.977; Sn, 94%; Sp, 90%	[327]
PBMC	PBIScore, AFP/CA19‐9	633 HD; 790 HCC, 341 benign hepatic diseases; 376 PDAC, 208 benign pancreatic diseases	RF	HCC AUC, internal validation cohort 0.97, external validation cohort 0.96; PDAC AUC, internal validation cohort 0.98, external validation cohort 0.97	[214]
Protein	Tumor‐associated autoantibodies	Training dataset: 236 ESCC, 236 HD; Validation dataset: 131 ESCC, 131 HD	SVM	AUC, 0.83; accuracy, 0.77	[260]
Protein	Tumor‐associated autoantibodies	Internal validation set: 312 ESCC, 101 HD; External validation set 1: 237 ESCC, 134 HD; External validation set 2: 101 ESCC, 101 HD	Partial least squares generalized linear models (*plsRglm*)	AUC: internal validation set, 0.826; external validation set 1, 0.851; external validation set 2, 0.850	[328]
Protein	β‐hCG, PD‐L1, AFP, age	Breast cancer (*n* = 124), HD (*n* = 33)	Ensemble learning (voting classifier) based on RF, XGBoost, AdaBoost, SVM, LR	Accuracy, 97.93%	[263]
Protein	Cancer type specific differently expressed proteins	175 patients of 4 cancer types, 65 HD (training:validation = 8:2)	RF	Sn, 87.0%; accuracy, 73.8%	[262]
Prognostic assessment					
Protein	Total serum protein level, CA19‐9, C‐creative protein, etc.	Training cohort: PDAC (*n* = 203); Validation cohort: Stage IV PDAC with liver metastases (*n* = 22)	Random survival forest	Train cohort: C‐index 0.71; Validation cohort: C‐index 0.67	[329]
EVs	Raman spectrum characteristics	Primary glioblastoma, breast and lung metastasized brain cancer patients (*n* = 30)	CNN	Primary‐metastatic sample classification: AUC 0.988; Primary tissue of origin prediction: accuracy 97%	[174]
cfDNA	Differentially methylated CpG probes	Training cohort: 16 recurrence meningioma, 9 nonrecurrence; Validation cohort: 8 recurrence meningioma, 12 nonrecurrence	Random forest	C‐index 0.87	[77]
cfDNA	DNA methylation profiles	Nonmuscle‐invasive bladder cancer (*n* = 601) (training:validation = 2:1)	partDSA, SS‐RPMM	OS significantly differentiate among 3 groups partDSA (log‐rank *p* < 0.001), SS‐RPMM AUC, 0.91	[233]
ctDNA	Variants, variant allele fraction, dynamic changing rate	NSCLC (*n* = 181)	PROPHET	A median lead time of 299 days to radiological recurrence	[4]
Treatment monitoring					
CTCs	4 differentially expressed genes driven by DNA methylation	Colorectal cancer	CNN	100 and 28.21% response rate in benefit and no‐benefit patient groups after the immunotherapy	[330]
EVs	Raman spectrum characteristics of HER2^+^ EVs pre‐ and post‐2 cycles of Neoadjuvant therapy	HER2^+^ breast cancer (*n* = 56)	PCA–LDA–SVM	Prediction accuracy of effectiveness of the therapy, >0.94	[313]

Abbreviations: AUC, area under the curve; CNN: convolutional neural network; CNV, copy number variations; CRC, colorectal cancer; DL, deep learning; ESCC, esophageal squamous cell carcinoma; GBM, gradient boosting machine; GC, gastric cancer; GLM, generalized linear model; HCC, hepatocellular carcinoma; HD, healthy donors; LASSO, least absolute shrinkage and selection operator; LDA, linear discriminant analysis; LR, logistic regression; NSCLC, non‐small cell lung cancer; partDSA, partitioning deletion/substitution/addition algorithm; PCA, principal components analysis; PDAC, pancreatic ductal adenocarcinoma; PROPHET, Patient‐specific pROgnostic and Potential tHErapeutic marker Tracking; RF, random forest; Sn, sensitivity; Sp, specificity; SS‐RPMM, semi‐supervised recursively partitioned mixture model; SVM, support vector machine; TCR, T‐cell receptor; UCEC, uterine corpus endometrial carcinoma.

## 10 Conclusion

Peripheral blood biomarkers offer a minimally invasive, dynamic, and integrative perspective on solid tumors, markedly expanding the repertoire of tools available for cancer diagnosis, prognosis, and therapeutic monitoring. A comparative analysis of these biomarkers’ characteristics indicates their suitability for different clinical settings. However, widespread clinical translation remains constrained by several factors. Future research should prioritize standardizing detection methodologies, advancing technologies to enhance analytical performance, and developing multiplexed biomarker panels that harness the synergistic value of diverse biomarker types. With continued innovation and validation, peripheral blood biomarkers are poised to play a pivotal role in achieving earlier detection, more precise disease monitoring, and ultimately, improved clinical outcomes in precision oncology.

## Author Contributions

J.X. conceived the project. X.T. and M.T. wrote the original draft. J.X., X.T., and M.T. revised the paper. J.X. supervised the project. All authors have read and approved the final manuscript.

## Funding

The work was supported by the Key R&D Program of Zhejiang (grant number 2026C02A1110, awarded to J. Xu), the National Natural Science Foundation of China (grant number 82472891, awarded to J. Xu), Zhejiang Provincial Natural Science Foundation of China (grant number LZ24H160001, awarded to J. Xu), and the Medical Interdisciplinary Innovation Program 2024, Zhejiang University School of Medicine of China.

## Ethics Statement

The authors have nothing to report.

## Conflicts of Interest

The authors declare no conflicts of interest.

## Data Availability

The authors have nothing to report.
